# The gut microbiota is an emerging target for improving brain health during ageing

**DOI:** 10.1017/gmb.2022.11

**Published:** 2022-12-12

**Authors:** Marcus Boehme, Katherine Elizabeth Guzzetta, Caroline Wasén, Laura Michelle Cox

**Affiliations:** 1 Nestlé Institute of Health Sciences, Nestlé Research, Société des Produits Nestlé S.A., Lausanne, Switzerland; 2 APC Microbiome Ireland, University College Cork, Cork, Ireland; 3 Department of Anatomy and Neuroscience, University College Cork, Cork, Ireland; 4 Ann Romney Center for Neurologic Diseases, Harvard Medical School, Brigham and Women’s Hospital, Boston, MA, USA; 5 Department of Biosystems Science and Engineering, ETH Zurich, Basel, Switzerland; 6 Department of Biology and Biological Engineering, Chalmers University of Technology, Gothenburg, Sweden

**Keywords:** Gut microbiome, brain ageing, microbiota-gut-brain axis, probiotics, cognition, neurodegeneration

## Abstract

The gut microbiota plays crucial roles in maintaining the health and homeostasis of its host throughout lifespan, including through its ability to impact brain function and regulate behaviour during ageing. Studies have shown that there are disparate rates of biologic ageing despite equivalencies in chronologic age, including in the development of neurodegenerative diseases, which suggests that environmental factors may play an important role in determining health outcomes in ageing. Recent evidence demonstrates that the gut microbiota may be a potential novel target to ameliorate symptoms of brain ageing and promote healthy cognition. This review highlights the current knowledge around the relationships between the gut microbiota and host brain ageing, including potential contributions to age-related neurodegenerative diseases. Furthermore, we assess key areas for which gut microbiota-based strategies may present as opportunities for intervention.

## Introduction

Physiological changes that occur during ageing can contribute to a progressive decline in overall brain health including cognitive abilities. This impairment in brain health is observable in multiple age-related diseases, including mild cognitive impairment (MCI), Alzheimer’s disease (AD), Parkinson’s disease (PD) and multiple sclerosis (MS), and leads to a worsened quality of life for patients. There is an important difference between one’s chronologic age (time since birth) versus one’s biologic age, and differences in the rate of ageing, especially during midlife, can significantly impact future age-related decline (Elliott et al., 2021). Furthermore, biologic age in healthy elderly individuals can predict the development of dementia (Wu et al., 2021a). Recent studies suggest that peripheral changes in physiology may affect age-related cognitive decline and therefore represent an emerging therapeutic target to manage brain health in ageing. Moreover, gastrointestinal (GI) functions are disrupted during ageing, including weakened gut barrier function, altered intestinal neurotransmitter levels and altered intestinal immunity (Bosco and Noti, 2021; Saffrey, 2014). Changes in GI physiology culminate in alterations to the gut microbiota, which may in turn influence brain ageing.

Trillions of microorganisms reside in the GI tract and constantly survey, adapt and respond to their environment. Collectively known as the *gut microbiota*, these microbes have coexisted and coevolved with their host and are increasingly recognised for their contributions to maintaining the health of their host throughout the lifespan, including brain health. The gut microbiota can communicate bidirectionally with the brain through several mechanisms, including endocrinal, nervous, immune and microbial metabolite-driven pathways (Cryan et al., 2019b). Investigations into these pathways, along with the use of germ-free (GF) rodents, which completely lack all microbes, and direct gut microbiota manipulation, such as antibiotic treatment, faecal microbiota transplantation and microbiota administration, have enabled a deeper understanding of how the gut microbiota influences biological functions in its host, including in the brain. Critically, the gut microbiota influences the blood–brain barrier (Braniste et al., 2014), neurochemistry and cellular functions in the brain, such as immunity and neuroplasticity (Erny et al., 2015; Guzzetta et al., 2022; Mossad and Blank, 2021; Ogbonnaya et al., 2015; Parker et al., 2022; Rei et al., 2022; Scott et al., 2017; Spichak and Guzzetta, 2018) and has been preclinically shown to modulate cognitive function during ageing (Boehme, Guzzetta and Bastiaanssen et al., 2021; Mossad et al., 2021), and neurodegeneration (Erny et al., 2021). Taken together, the gut microbiota plays a crucial role in supporting healthy cognition and neurological functions of its host, including active participation in important aspects of brain ageing.

While the composition of the gut microbiota is generally understood to be relatively stable during early- and mid-adulthood, community stability appears to be uprooted in later stages of ageing, although the effects of ageing on gut microbiome diversity appear mixed, perhaps due to factors such as health and diet. For instance, elderly patients in care homes displayed an overall reduction in faecal microbial diversity, which also associated to worsened health status of the individual, while centenarians living amongst the general population had distinguishably increased alpha diversity, due to higher relative abundance of subdominant taxa accompanied with reduced relative abundances of core taxa (Biagi et al., 2016, 2017; Claesson et al., 2012; Fanli Kong et al., 2016; Wu et al., 2019). These conflicting results suggest that ageing leads to divergences in the gut microbiome which are dependent on the community sampled, and which likely contribute to an increased microbiome uniqueness overserved in ageing (Wilmanski et al., 2021). Understanding the causal relationships between gut microbiome trajectory and healthy brain ageing may allow for the discovery of novel microbiota-driven therapies for maintaining brain health during ageing.

## Biological ageing drives changes to the gut microbiome

### The gut microbiome during ageing

Following birth, the composition of the gut microbiome is highly dynamic, and gradually increases in stability with the development of the adaptive immune system, weaning onto a solid food diet and standardisation of lifestyle factors in early adulthood (Koenig et al., 2011). While the composition of the gut microbiome is relatively stable during early- to mid-adulthood if unperturbed, as an individual grows older, the gut microbiota enters a period of increased volatility and distinct shifts occur in the diversity of genera and functional capacity (Badal et al., 2020; Claesson et al., 2011). Ageing-associated alterations to microbial diversity and composition of the gut microbiome have been observed in multiple species, including flies (eg., *Drosophila*), worms (eg., *Caenorhabditis elegans*), rodents, dogs, monkeys (Ambrosini et al., 2019; Dinić et al., 2021; Kubinyi et al., 2020; Lee et al., 2019; Pallikkuth et al., 2021; Scott et al., 2017; van der Lugt et al., 2018; Wei et al., 2021) and in humans (Biagi et al., 2010; Claesson et al., 2012; Jeffery et al., 2016; Leite et al., 2021). This may be due to a multitude of biological and environmental factors, including immunosenescence (Bosco and Noti, 2021), altered physiology of the gastrointestinal tract (Soenen et al., 2016), development of age-related diseases, and in humans, increased exposure to medication and altered diet associated with long-term care facilities (Claesson et al., 2012).

In humans, characteristics of ageing within the gut microbiome begin to appear in mid-to-late adulthood (Wilmanski et al., 2021), though the exact timepoint for this shift may vary based on genetic, environmental and lifestyle factors which are unique to an individual. In general, ageing is associated with a decline in core microbial genera, increased bacterial community uniqueness, altered microbial diversity and altered functional ability of the gut microbiota (Claesson et al., 2011, 2012; Ghosh et al., 2022a,b; Wilmanski et al., 2021). Critically, there is noticeable variation between studies examining the gut microbiome within different older populations, which may be due to dietary, cultural, environmental or geographical differences that can influence the gut microbiome, or due to differences in microbiome analysis methodology (Biagi et al., 2010, 2017; Claesson et al., 2012; Odamaki et al., 2016; Salazar et al., 2017; Wilmanski et al., 2021). For instance, the genus *Bacteroides* and other genera within the family *Bacteroidales* including *Alistipes* and *Parabacteroides* have been observed at higher levels in people aged 65+ compared to younger, healthy adults (Claesson et al., 2011). However, other studies indicate that ageing leads to a decrease in *Bacteroidaceae*, the family that contains *Bacteroides*, along with a reduction in *Faecalibacterium* and *Lachnospiraceae*, and an increased abundance of *Akkermansia*, as recently reviewed by Badal et al. (2020).

These differences in study outcomes suggest that it is currently impossible to pinpoint exactly when a person’s gut microbiome shifts to that of an ‘elderly’ state. Nonetheless, an ageing-associated drift in the gut microbiome was not as prevalent in less healthy individuals, suggesting that the shift in the microbiome occurring during ageing may be beneficial for and predictive of host health (Wilmanski et al., 2021). Furthermore, retention of a higher prevalence of *Bacteroides* or a low microbial uniqueness was associated with a higher risk of mortality in a 4-year follow up (Wilmanski et al., 2021). This research highlights that the microbiota may act as a novel marker for lifespan in elderly humans with specific bacterial genera potentially playing important roles.

### Extreme ageing shows unique microbial signatures

It is important to consider the difference between biologic ageing that leads to physiologic decline versus healthy ageing that supports longevity. While the gut microbiome signatures associated with increased mortality uncovered by Wilmanski et al. (2021) may be detrimental, there may be beneficial microbes unique or more abundant in healthy, long-living individuals that could support the health of their host (Wilmanski et al., 2021). Indeed, unique microbial signatures appear as an individual ages and are highly apparent in centenarians (individuals who have lived for 100 years or more), though differences in bacterial genera appear to vary depending on the culture and region in which the assessment occurred (Biagi et al., 2016; Kim et al., 2019a; Kong et al., 2016; Odamaki et al., 2016; Sato et al., 2021; Tuikhar et al., 2019; Wilmanski et al., 2021). In a well-characterised Italian cohort, the gut microbiome of centenarians had higher abundance of several taxa such as *Akkermansia* and *Christensenellaceae*, some of which have previously been associated with health, suggesting they may contribute to the maintenance of health during ageing (Biagi et al., 2016). Meanwhile, other research found that *Ruminococcaceae*, *Lachnospiraceae* and *Bacteroidaceae* families decreased with advancing age (Biagi et al., 2016; Pu et al., 2021). In another investigation in South Korea, *Akkermansia*, *Collinsella*, *Clostridium* and *Christensenellaceae* were increased in the faecal microbiome of centenarians, while there was a decreased abundance of *Faecalibacterium* and *Prevotella* (Kim et al., 2019b). This was associated with a greater predicted ability of the gut microbiome to contribute to glycosphingolipid biosynthesis, various N-glycan biosynthesis and the phosphatidylinositol signalling pathway (Kim et al., 2019a). While several studies have reported different findings regarding the composition of the gut microbiome and ageing (Badal et al., 2020; Ghosh et al., 2022a), synergies between clinical studies may highlight potential health-promoting or health-degrading properties of specific bacteria, which could inspire investigations into whether these bacteria may also support cognitive health during extreme ageing (for further reading on this topic, see Ghosh et al., 2022b).

It is tempting to speculate that longevity-associated bacterial taxa might be involved in the establishment of a longevity-promoting environment within the ageing host, and thus contribute to supporting the extreme limits of ageing within the human life, perhaps by buffering an individual from environmental challenges. Indeed, initial research involving the African turquoise killifish (*Nothobranchius furzeri*) demonstrated a causal role of the gut microbiota in longevity; transferring microbiota from young fish into middle-aged fish improved their lifespan and motor behaviour and was accompanied by distinct transcriptional changes in intestinal immunity (Smith et al., 2017). Furthermore, in a mouse model of progeria, which is a condition of accelerated ageing wherein gut microbiome is also altered, progeroid mice that received the faecal microbiota from wild-type mice showed a noticeable increase in lifespan and health span (Barcena et al., 2019). While whole faecal microbiota transplantation (FMT) has shown success in improving lifespan in these models, there is currently no evidence demonstrating whether FMT has a beneficial impact on human lifespan. Moreover, FMT is an intensive clinical procedure. Therefore, the use of individual probiotic bacteria, which can confer health benefits in ageing, may prove to be a better strategy for intervening on the ageing microbiome. Indeed, single strains of bacteria may hold the key for lifespan extension; administration of the bacterium *Akkermansia muciniphila* alone was sufficient to exert similar effects on extending lifespan in progeroid mice (Barcena et al., 2019). Whether these microbiota-targeted interventions could impact brain health during advanced ageing was not assessed in these studies, but other research has demonstrated a protective effect of *Akkermansia* in neurologic diseases including models of multiple sclerosis, amyotrophic lateral sclerosis, epilepsy and Alzheimer’s disease (Blacher et al., 2019; Cox et al., 2021; Liu et al., 2019; Olson et al., 2018; Ou et al., 2020), suggesting there could be a benefit of elevated *Akkermansia* in ageing. Alternatively, certain bacteria may be harmful rather than helpful. Research involving germ-free mice has shown that they live roughly 17 per cent longer than their specific pathogen free peers, suggesting that there may be consequential interactions between bacteria and their host that accelerate ageing (Tazume et al., 1991). Therefore, the elimination of these ageing-related potentially pathogenic microbes, perhaps through select antibiotic usage or CRISPR knockout of individual genes or pathogens, presents another potential intervention strategy. However, these strategies are currently limited by a lack of scientific understanding of what makes the bacteria behave pathogenically.

## The gut microbiota actively contributes to brain health during ageing

### The microbiota and the ageing brain: establishing connections

**Figure 1.**
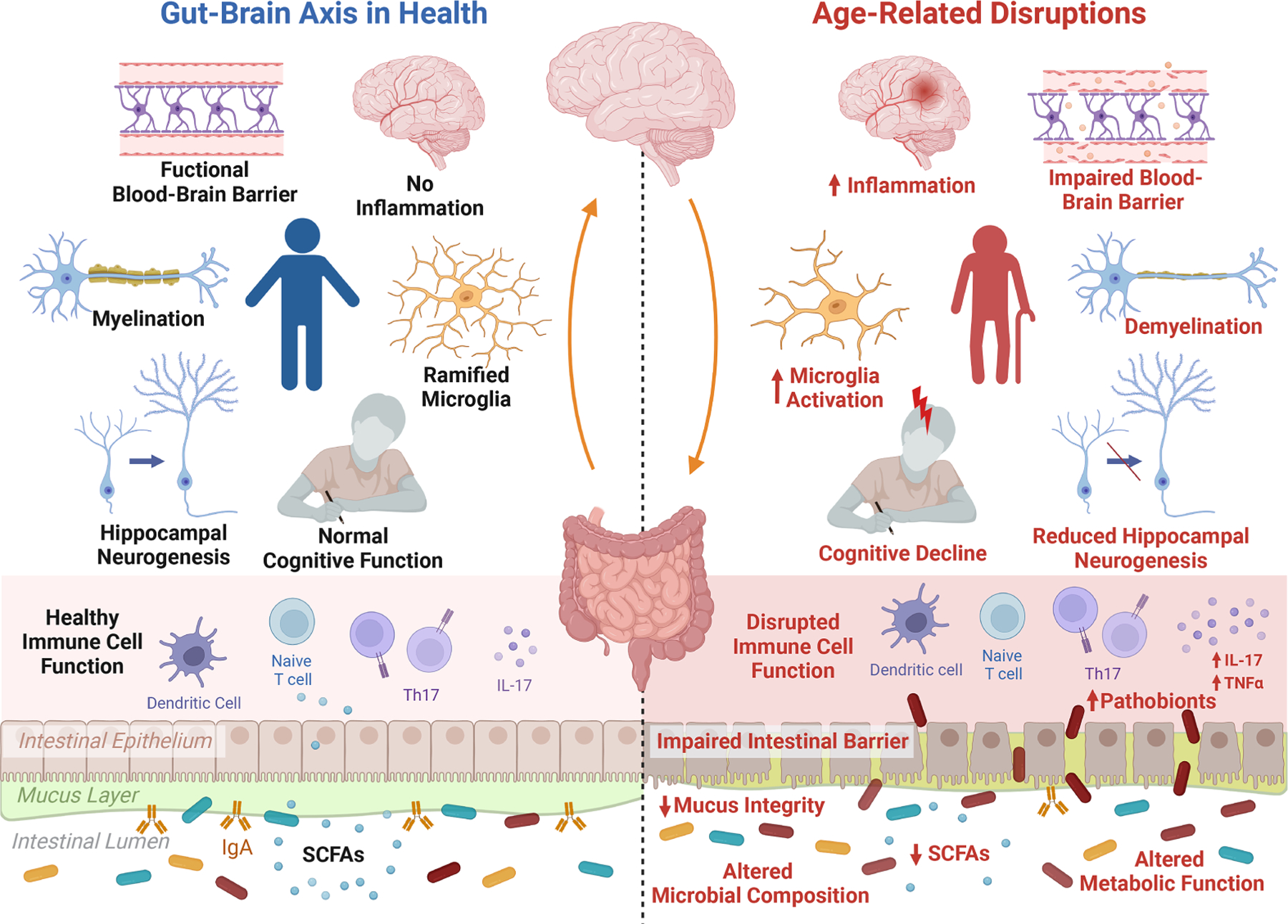
The microbiota–gut–brain axis during ageing. The process of ageing initiates distinct alterations in the gut, brain and signalling pathways. These manifest in alterations in the gut microbiota composition and metabolic function, including a reduction in short-chain fatty acids (SCFAs), depleted mucus integrity and impaired intestinal barrier function, allowing for increased invasion of pathobionts from the intestinal lumen. Furthermore, low-grade inflammation and immunosenescence occurs during ageing, disrupting the function of immune cell populations and leading to systemic inflammation triggered by increased pro-inflammatory cytokines, such as interleukin (IL)-17. This inflammatory state is also noticeable within the ageing brain, and is related to increased activation of microglia, the brain’s primary immune cell. Furthermore, ageing is associated with a decline in blood–brain barrier integrity, demyelination, a reduction in hippocampal neurogenesis and increased inflammation resulting in more microglia activation, which all in all may contribute to ageing-related cognitive decline. Created with BioRender.com.

The ageing brain is characterised by changes on multiple levels ranging from cellular and morphological to functional changes (Peters, 2006). Systemic low-grade inflammation occurs and can lead to increased permeability of the blood–brain barrier which can trigger microglia activation, cause neuroinflammation and can increase the production of reactive oxygen species which can lead to increased oxidative stress and mitochondrial dysfunction (Peters, 2006). Ageing can also induce a decline in neuronal volume and alterations in neurotransmitter levels such as dopamine, serotonin and gamma-aminobutyric acid (GABA). This can trigger impaired synaptic plasticity and impaired neurogenesis and can cause functional deficiencies such as cognitive impairments (Figure 1). Following adulthood, the volume of the brain gradually shrinks, along with diminished grey and white matter in distinct brain areas in a sex-dependent manner such as cortical and subcortical areas, which are implicated in cognitive processes (Murphy et al., 1996; Peters, 2006). Further changes in vasculature and diffusion can occur, which can underly neurodegenerative diseases and stroke (Lendahl et al., 2019).

While the impact of ageing on the mammalian (Fransen et al., 2017; Thevaranjan et al., 2017; van der Lugt et al., 2018) and human (Biagi et al., 2016; Claesson et al., 2012; Jeffery et al., 2016; Leite et al., 2022; Wilmanski et al., 2021) gut microbiome is well established, the impact of the gut microbiota on brain ageing has only recently been investigated, and relies dominantly on preclinical evidence and association analysis derived from small clinical trials.

Studies utilising FMT have demonstrated that the gut microbiota from aged individuals has the capacity to hinder cognitive performance and neurobiological phenotypes when transferred to younger individuals. For instance, transplanting microbes from aged and diseased models to young mice has been shown to impair learning, memory and neuroplasticity in recipient young mice (D’Amato et al., 2020; Kim et al., 2021; Li et al., 2020). This coincided with alterations in microbial metabolites such as short-chain fatty acids (SCFAs; D’Amato et al., 2020) which have previously been shown to regulate host microglial maturation and function (Erny et al., 2021). Moreover, using humanised models by transplanting FMT from older subjects with Alzheimer’s disease into microbiota-depleted naïve adult rats showed impairment in hippocampal neurogenesis accompanied with memory deficits, which correlated with clinical cognitive scores (Grabrucker et al., 2022). Furthermore, young mice who received gut microbiota from aged donors suffered increased rates of mortality following ischemic stroke, along with increased levels of pro-inflammatory plasma cytokines and impaired motor strength (Spychala et al., 2018). Conversely, aged mice who were colonised with the microbiota from young donor mice had increased survival and improved recovery post-stroke (Spychala et al., 2018), demonstrating the functional differences between microbiota derived from young or aged individuals in influencing brain recovery following trauma.

The age of the FMT recipient also appears to have a strong influence on how an individual responds to an FMT. Counterintuitively, young germ-free (GF) mice who received microbiota from old mice demonstrated increased hippocampal neurogenesis and signs of pro-longevity compared to those who received FMT from young mice, while the same effects did not occur in aged GF mice (Kundu et al., 2019). Hippocampal neurogenesis is influenced by the gut microbiota (Guzzetta et al., 2022; Ogbonnaya et al., 2015), and is well characterised to decline with ageing (Kozareva et al., 2019), suggesting there are underlying age-driven factors that may supersede how an individual responds to a microbial community (Wilmanski et al., 2022). Overall, these studies highlight that there are functional differences in the gut microbiota of aged individuals which may be contributing to the decline in cognition and altered neurobiology that occurs during ageing.

Fascinatingly, the gut microbiota from young mice appears to harness properties that enable it to rejuvenate aspects of brain ageing when transferred into aged mice. Two studies recently confirmed similar findings, wherein FMT from young mice to aged mice improved ageing-related deficits in memory and learning ability (Boehme, Guzzetta, Bastiaanssen et al., 2021; Mossad et al. 2021). Microbiota from young mice restored age-related changes in peripheral and hippocampal immune responses and reversed age-related alterations in hippocampal transcriptional profiles and metabolites (Boehme, Guzzetta, Bastiaanssen et al., 2021), suggesting potential mechanisms by which the gut microbiota from young mice improve cognitive performance by modulating immune and metabolic pathways. In this regard, the microbially-derived metabolite δ-valerobetaine, which is increased in aged mice and humans, was shown to directly impair learning and memory abilities, and was reduced in aged mice following FMT from young donor mice (Mossad et al., 2021). Relatedly, other gut microbiota-derived metabolites which are linked to age-related shifts in the gut microbiota and are increased in aged humans indicate that specific microbially-derived metabolites can impair cognitive abilities during ageing. For example, isoamylamine supplementation induced cognitive dysfunction by triggering microglia cell death in young mice (Teng et al., 2022). Meanwhile, N6-carboxymethyllysine administration lead to oxidative stress and mitochondrial damage in microglia in young and aged mice, although a starker effect was evident in young mice when N6-carboxymethyllysine was administered intraperitonially rather than by oral gavage whereas the delivery method was insignificant in aged mice, suggesting a protective role of the intestinal barrier in minimising damage caused by harmful ageing-related microbially-derived metabolites (Mossad et al., 2022).

On the other hand, it is currently unclear whether ageing-driven changes in the gut are protective or harmful to cognitive health in ageing. For instance, research by Wilmanski et al. (2021) found that a higher gut microbiome uniqueness score correlated to several health metrics in elderly individuals, whereas a lower gut microbiome uniqueness score was associated with earlier mortality. This could suggest that ageing-related changes in the gut foster increased uniqueness in the microbiome which may in turn prevent or slow the progression of ageing. Therefore, there may exist key bacteria that are increased in healthy ageing, and the inability of these probiotic bacteria to sustain and function in the unhealthy ageing gut may underlie consequences of ageing. With this theory in mind, we should not assume that FMT from young to aged individuals provides only beneficial effects, as this procedure could also influence the existing, sensitive microorganisms supporting healthy ageing. In order to fully understand the beneficial and/or harmful impacts of FMT on the ageing brain, more research needs to be conducted to thoroughly investigate how FMT may impact broad aspects of health in ageing.

### The gut microbiome during brain ageing and in age-related neurodegenerative diseases

Accumulating evidence is revealing associations between the gut microbiota and neurodegenerative diseases associated with ageing, including mild cognitive impairment (MCI), Alzheimer’s disease (AD), Parkinson’s disease (PD) and multiple sclerosis (MS) (Alkasir et al., 2017; Cox et al., 2021; Guo, 2021; Romano et al., 2021; Saji et al., 2019; Vogt et al., 2017). In addition to alterations in the microbiota in human populations, studies involving preclinical disease models highlight the potential causal roles which the gut microbiota plays in host neurodegenerative diseases (Berer et al., 2017; Dodiya et al., 2021; Harach et al., 2017; Perez-Pardo et al., 2017; Sampson et al., 2016). The interaction of the microbiota in AD, PD and MS has been extensively studied (Kincaid et al., 2021; Mirza et al., 2020; Tansey et al., 2022). Thus, we will focus our review on highlighting key findings related to the contribution of the ageing gut microbiota to these diseases and healthy brain ageing, and the current evidence for the use of gut microbiota-targeted therapeutics.

#### Alzheimer’s disease and mild cognitive impairment

The most common age-related dementia, AD, is estimated to affect about 57 million individuals worldwide with projections to 152 million in 2050 (Nichols et al., 2022) while pre-dementia states such MCI has a prevalence of about 16 per cent amongst elderly subjects in the USA, with amnestic MCI, which has a high likelihood to progress to AD, as the most common type (Petersen et al., 2010). Notably, the gut microbiota of AD patients living in the USA has been shown to contain reduced relative abundance of *Firmicutes* and *Bifidobacterium*, and higher levels of *Bacteroidetes*, including *Bacteroides* (Vogt et al., 2017). In a Chinese cohort AD patients had lower levels of *Lachnospira*, *Bacteroides* and *Ruminiclostridium_9*, as well as an increased abundance of *Prevotella* compared with healthy, age-matched peers (Guo, 2021). Similarly, a Japanese cohort examining elderly patients with mild cognitive impairment but no dementia, versus healthy elderly found higher prevalence of *Bacteroides* in patients with cognitive decline (Saji et al., 2019), though others have observed only *Lachnospira* was significantly lower in patients with mild cognitive impairments (Guo, 2021). Additional research into gut microbiota of individuals ranging from healthy ageing over MCI to AD has revealed that bacterial genera that were differentially abundant in AD were also different in MCI, suggesting that changes in the gut microbiome might precede AD onset (Li et al., 2019).

While clinical mechanistic evidence is largely lacking, two case studies reveal a glimpse of hope for the direct potential of the gut microbiota to alleviate symptoms of dementia in AD. Fascinatingly, an 82-year-old man with AD who was administered FMT to treat *Clostridioides difficile* infection showed improved cognitive score in the Mini-Mental State Examination (MMSE) from MCI levels to healthy cognition at his 2-month follow-up visit (Hazan, 2020). Similarly, a 90-year-old woman suffering from AD treated with FMT for *C. difficile* displayed a marked improvement in several cognitive function tests within 3 months (Park et al., 2021). This clinical FMT literature, albeit limited by sample size, supports the translatability of previous pre-clinical findings wherein FMT from young C57BL/6 wild-type male mice rejuvenated aspects of cognition when transplanted into aged male C57BL/6 mice (Boehme, Guzzetta, Bastiaanssen et al., 2021). Furthermore, FMT from wild-type mice into male APP/PS1 transgenic mice, a genetic mouse model of AD, led to reductions in memory impairment, Aβ accumulation, synaptic dysfunction and neuroinflammation (Sun et al., 2019), while FMT from an additional genetic AD mouse model known as 5xFAD mice into naïve C57BL/6 mice lead to decreased hippocampal neurogenesis and cognitive impairment (Kim et al., 2021). In addition to the transfer of the full microbiota between animals, attempts to modulate AD pathology by administrating specific AD-associated bacteria have also been made. Increasing levels of *Bacteroides* were reported in ageing Tg2576 transgenic mice and correlated to the amyloid plaque levels in their brains, and weekly administration of *Bacteroides fragilis* between 2 and 5 months of age increased amyloid plaque burden in APP/PS1 mice (Cox et al., 2019).

Furthermore, there are hypotheses that the gut microbiota contributes to the development of AD, which is supported by preclinical evidence involving antibiotic administration to rodent models of AD. For instance, cocktail of antibiotics given to three different amyloid mouse models of AD (APP/PS1, 5xFAD and App^NL-G-F^) from early age reduced the amyloid plaque load observed later in life in male mice (Guilherme et al., 2021; Kaur et al., 2021; Mezö et al., 2020; Minter et al., 2016), while no difference has been observed in females. It was further demonstrated that it was sufficient to administer the antibiotic cocktail early in life (pre-weaning) to observe lasting alterations in the gut microbiome and reduced amyloid pathology later in life (Minter et al., 2017). The administration of individual antibiotics, however, did not reduce the accumulation of amyloid plaques (Dodiya et al., 2020). The antibiotic cocktail administered early in life reduced alpha-diversity in the gut but led to increased relative abundance of *Akkermansia* in the APP/PS1 model (Minter et al., 2017). Interestingly, mice given antibiotics in early life had lasting alterations to peripheral and central immunity, with increased proportions of regulatory T cells. Moreover, microglia appears critical for inducing this response; mice with depleted microglia did not have reduced plaque levels after treatment with the antibiotic cocktail, although microglia depletion also slightly reduced plaque burden (Dodiya et al., 2021). Antibiotic treatment in the 5xFAD model of AD improved spatial and recognition memory performance, which was also demonstrated in GF mice (Mezö et al., 2020). Treatment with antibiotics from the timepoint of weaning also reduced anxiety in wild-type mice that were subjected to an intracerebral injection of amyloid peptides to simulate the onset of AD at 80 days of age (Mosaferi et al., 2021). Overall, this growing body of evidence strongly implicates the gut microbiota in the pathology of AD, as well as a potential target for novel therapeutics.

#### Parkinson’s disease

Parkinson’s disease (PD) is the second most common age-related neurodegenerative disease, and may have unique influences from the gut microbiota initiating disease pathogenesis in the gut (Yang et al., 2019). Mouse studies suggest that the underlying mechanism causing the death of dopaminergic neurons in the striatum, a brain region that controls motor behaviour, is caused by the propagation of misfolded alpha-synuclein along the vagus nerve (Kim et al., 2019a). Indeed, PD patients show gastrointestinal symptoms long before the onset of PD (Sung et al., 2014). A Danish study investigated the risk for PD in subjects who underwent vagotomy back in the 1970s and 1980s when it was used as a therapy to treat peptic ulcer disease and found that truncal vagotomy, which cuts the entire nerve, was associated with a decreased risk to develop PD (Svensson et al., 2015). This observation was confirmed two years later in a Swedish cohort (Liu et al., 2017). These epidemiological findings support the view that PD initially commences in the gut and not the brain, and strongly implicates a role of the vagus nerve in the pathogenesis of PD. A recent preprint now suggests that inflammatory bowel disease (IBD) is linked to PD suggesting that anti-inflammatory therapies targeting the gut may be preventive for PD later in life (Espinosa-Oliva et al., 2022). In animal models of PD, transfer of microbiota from new-onset treatment-naïve PD patients worsened motor function in alpha-synuclein overexpressing mice (Sampson et al., 2016), and depleting the microbiota with antibiotics or by germ-free status improved motor symptoms, which was linked to changes in microglia function. This study supports the potential role of the gut microbiota in contributing to PD. In a study of Pink1^−/−^ mice, early-life infection with *Citrobacter rodentium* increased motor dysfunction and disease pathogenesis, which was linked to increased mitochondrial specific cytotoxic CD8 T cells in the brain (Matheoud et al., 2019). While PD is considered an age-related disease, this study suggests that immunologic programming in infancy may affect PD pathogenesis. Thus, gut-microbiota interactions throughout lifespan may be important determinants of age-related neurologic disease.

#### Multiple sclerosis

Multiple sclerosis (MS) is a demyelinating neurodegenerative disease, with a typical age of onset in the 20s to 40s, and ageing can play an important role in disease progression (Dobson and Giovannoni, 2019). The majority of MS patients develop a relapsing remitting form of the disease (RRMS), while a smaller proportion of patients will initially present with a progressive form of the disease (primary progressive MS, PPMS (Lassmann, 2018). Throughout the disease course, many RRMS patients will transition to a secondary progressive disease course (SPMS), and ageing is one of the largest risk factors for developing SPMS (Lassmann, 2018). Because progressive MS is more refractory to treatment and is associated with higher disability and quality of life, it is critical to understand whether the host-microbiome interactions in ageing affect this transition. Patients diagnosed with progressive MS show increased *Akkermansia, Clostridium bolteae* and *Ruthenibacterium lactatiformans* along with reduced levels of *Blautia wexlerae*, *Dorea formicigenerans* and *Erysipelotrichaceae CCMM* and altered microbial β-diversity (Cox et al., 2021). The abundance of *Clostridium* species was associated with worsened disability status on the Expanded Disability Status Scale, suggesting a potentially detrimental role. Interestingly, although *Akkermansia* was elevated in progressive MS, higher abundance of *Akkermansia* correlated with lower disability, and was demonstrated to ameliorate aspects of disease pathology in a mouse model of multiple sclerosis, perhaps through its ability to reduce RORγt+ and IL-17–producing γδ T cells (Cox et al., 2021). Therefore, it might be that some microbes act protectively against the progression of neurodegenerative disease, and that the absence of these species could be detrimental, although further research must be conducted in order to conclude this. In an animal model of MS known as the experimental autoimmune encephalomyelitis (EAE) model, treatment with antibiotics ameliorates disease and GF mice resist EAE, suggesting that the microbiota can contribute to diseases pathogenesis. Interestingly, the colonization of mice with the putative probiotic species Lactobacillus reuteri exacerbated EAE susceptibility, likely through L. reuteri’s ability to metabolize dietary tryptophan into active metabolites which can induce IL-17 production by binding to the aryl hydrocarbon receptor on T cells (Montgomery et al., 2022). Furthermore, transferring microbiota from MS patients into GF mice worsened EAE (Berer et al., 2017; Cekanaviciute et al., 2017). In terms of ageing, recent studies have shown that mice show a more progressive form of MS when they are older (Cahill et al., 2019), raising the question if age-related changes in the gut microbiota may drive MS disease progression.

### Potential mechanisms for microbiota–gut–brain signalling in ageing

The mechanism(s) of action underlying microbiota–gut–brain communication in ageing-related cognitive decline remain elusive. Given the increased inflammation and immunosenescence which occurs during ageing, it is probable that alterations to microbiota-immune signalling contribute to differences in host brain ageing. Indeed, recently literature has demonstrated that some ageing-induced alterations to gut-associated immunity were reversed following FMT from young to aged mice (Boehme, Guzzetta, Bastiaanssen et al., 2021). This FMT also resulted in decreased microglia soma area and reversed the age-associated increase in proinflammatory-associated microglial genes, which indicates decreased immune activation in the brain following FMT from young mice into aged mice (Boehme, Guzzetta, Bastiaanssen et al., 2021). Contrarily, this same study found that old mice who received microbiota from aged-matched mice had elevated plasma levels of the anti-inflammatory cytokine IL-10, which was not the case when old mice received FMT from young mice. This difference in plasma IL-10 could suggest that either transferring microbiota from young to aged mice may not only confer health benefits, or alternatively indicates that the inflammatory phenotype observed in old animals who received agematched FMT was no longer responsive to suppression by heightened IL-10.

Ageing-associated gut microbiota was previously demonstrated to play a key role in host inflammageing, suggesting the potential for microbiota-driven changes in gut–brain axis communication during ageing. Aged GF mice have lower levels of systemic inflammatory cytokines, including IL-6 and TNFα and increased macrophage ability to phagocytose bacteria compared to conventional aged mice, suggesting that the microbiome plays an important role in both inflammageing and immunosenescence (Thevaranjan et al., 2017). Furthermore, administering FMT from aged mice into young mice transferred the ageing-related phenotypes of inflammation (increased systemic IL-6 and TNFα), immune dysfunction and impaired gut barrier integrity, and was linked to a reduction of specific bacterial taxa such as *Akkermansia* (Fransen et al., 2017; Thevaranjan et al., 2017), directly implicating the gut microbiota as a causative contributor to inflammageing. Furthermore, when microbes were transferred from young to aged mice via heterochronic FMT, the ageing-induced deficiency in the Peyer’s patch germinal cell reaction, a key compartment of intestinal immunity, was rescued (Stebegg et al., 2019). This work demonstrated that the age-associated deficiency in intestinal stem cell activity was reversible by the gut microbiome by possibly providing them with the appropriate stimuli (Stebegg et al., 2019). While accumulating preclinical evidence strongly implicates the gut microbiota in regulating host longevity, health span and immunity during ageing, whether the gut microbiota regulates host brain ageing warrants further study.

Monocytes and microglia express stimulatory receptors that are activated by microbe-associated molecular patterns (MAMPs) and other small molecule secreted metabolites. These molecules are constantly produced by gut bacteria and shape host immunity (Chu and Mazmanian, 2013). Some studies suggest that bacterial components originating from the gut can be found in the human brain under pathological conditions. For example, a small study in six subjects with AD detected lipopolysaccharide (LPS) in the human hippocampus and neocortex (Zhao et al., 2017). Meanwhile, the bacterially-derived metabolite trimethylamine-N-oxide has been measured in human cerebrospinal fluid (Rio et al., 2017). Furthermore, a role of the oral microbiome in triggering neurodegenerative diseases such as Alzheimer’s disease is increasingly discussed (Shoemark and Allen 2015). For instance, toxins called gingipains from the oral bacteria *Porphyromonas gingivalis* have been identified in the brains of AD patients (Dominy et al., 2019). GF wild-type mice, or mice treated with high-dose antibiotics to nearly eliminate the microbiota had impaired maturation of microglia, which could be partially restored by orally administered bacteria-derived SCFAs (Erny et al., 2015). GF APP/PS1 mice, a model of AD, had lower amyloid beta (Aβ) protein levels, demonstrating that bacterial-derived metabolites can affect amyloid load (Harach et al., 2017; Minter et al., 2016). Colonising GF APP/PS1 mice with the microbiome of a conventionally raised APP/PS1 mouse increased Aβ levels compared to those colonised with WT microbiota (Harach et al., 2017).

Recent literature demonstrated that bacterial-derived metabolite acetate regulates the function of microglia in the brain. In the 5xFAD mouse model of AD, oral administration of acetate to GF mice promoted a pro-inflammatory phenotype in cortical microglia, with reduced phagocytosis of amyloid plaques leading to accelerated pathology (Erny et al., 2021). In contrast, work from a group in Japan showed that acetate partially counteracted cognitive impairment in another mouse model of AD in which Aβ was injected into the hippocampus of SPF mice (Kobayashi et al., 2017). In addition to acetate, it has been shown that supplementation with the second major short-chain fatty acid, butyrate, decreased neuroinflammation in aged mice (Matt et al., 2018). Another preclinical study found that when administered even at a late stage of Alzheimer’s disease-related pathology, butyrate supplementation improved associative memory in APP/PS1–21 mice (Govindarajan et al., 2011). The exact mechanism by which the gut microbiota exerts effects in AD through SCFAs and altered inflammation remains unknown, but potentially involves altered age-related pro-inflammatory monocyte trafficking to the brain (van de Wouw et al., 2019), as microglia are not known to express receptors for SCFAs (Erny et al., 2015). Furthermore, butyrate has been shown to affect the release of serotonin and gut hormone release in the enteric nervous system which can stimulate the vagus nerve and can trigger endocrine signalling, which can impact brain function (Stilling et al., 2016).

In addition to SCFAs, other microbially-derived metabolites have also been implicated in brain health during ageing. As discussed previously, the microbially-produced metabolite δ-valerobetaine is more abundant in older animals and was found to impair learning and memory abilities (Mossad et al., 2021). Meanwhile, FMT from young mice into aged recipient mice lowered levels of δ-valerobetaine systemically and in the brain, and improved learning and memory performance (Mossad et al., 2021). Using data from the UK Biobank, the authors showed that increasing human age is associated with an increase in systemic δ-valerobetaine levels. Interestingly, supplementation of δ-valerobetaine to GF and conventional mice enlarged their visceral fat mass, and systemic levels of this metabolite correlate with visceral adipose tissue mass in humans (Liu et al., 2021), a critical factor for dementia later in life (Cereda et al., 2007), suggesting δ-valerobetaine as a potential target for future therapies.

Meanwhile, indoles, which include a range of microbially-produced metabolites of dietary tryptophan, have been implicated to play a critical role in ageing. Through binding to the aryl hydrocarbon receptor, indoles can act locally on gut inflammation but also in the brain, as have been shown to modulate neuroinflammation through microglia control of astrocytes (Rothhammer et al., 2018). Furthermore, indoles in blood plasma have been positively associated with gut microbiome uniqueness (Wilmanski et al., 2021). Of note, *Bacteroides* species produce indoles, and high level of *Bacteroides* was linked with increased mortality in 85+ individuals (Wilmanski et al., 2021).

Accumulating clinical evidence suggests a link between bile acids and dementia (MahmoudianDehkordi et al., 2019; Varma et al., 2021; Wang et al., 2020). By combining transcriptomics analyses with a metabolic network analysis approach, researchers of the Alzheimer’s disease metabolomics consortium found that bile acid synthesis differs in AD compared to cognitively healthy individuals (Baloni et al., 2020). Fascinatingly, the presence of some of these bile acids measured in the brain cannot be explained by locally expressed enzymes, which suggests that they may be derived from the gut microbiome (Baloni et al., 2020). Preclinically, metabolomic analyses point to a role of secondary bile acids as a potential mechanism in rebalancing disturbed gut microbiota associated with an improvement in health and lifespan in progeria mice following FMT (Barcena et al., 2019). Interestingly, a recent study found that the secondary bile acid, isoallolithocholic acid, is increased in centenarians (Sato et al., 2021) and has been shown to influence host T-cell function by increasing the differentiation of Treg cells through increasing mitochondrial activity (Hang et al., 2019) though whether secondary bile acids can directly exert effects on the brain is currently unknown. Modulation of secondary bile acid metabolism might be a novel strategy to modulate brain ageing and may impact cognition in ageing.

The vagus nerve plays a fundamental role in microbiota–gut–brain axis signalling (Fulling et al., 2019). Intriguingly, a recent study found that an increase in vagal activity was able to mitigate the adverse effects of FMT from aged mouse donors on hippocampal function in young mice (Rei et al., 2022). Initial clinical data suggests that stimulation of the vagus nerve may improve some aspects of quality of life under specific conditions in elderly subjects (Bretherton et al., 2019), and improved walking speed and motor function in subjects with PD which was associated with a decrease in inflammatory biomarker and an increase in the myokine BDNF (Mondal et al., 2021).

## Strategies for targeting the gut microbiome for better brain health during ageing

**Figure 2.**
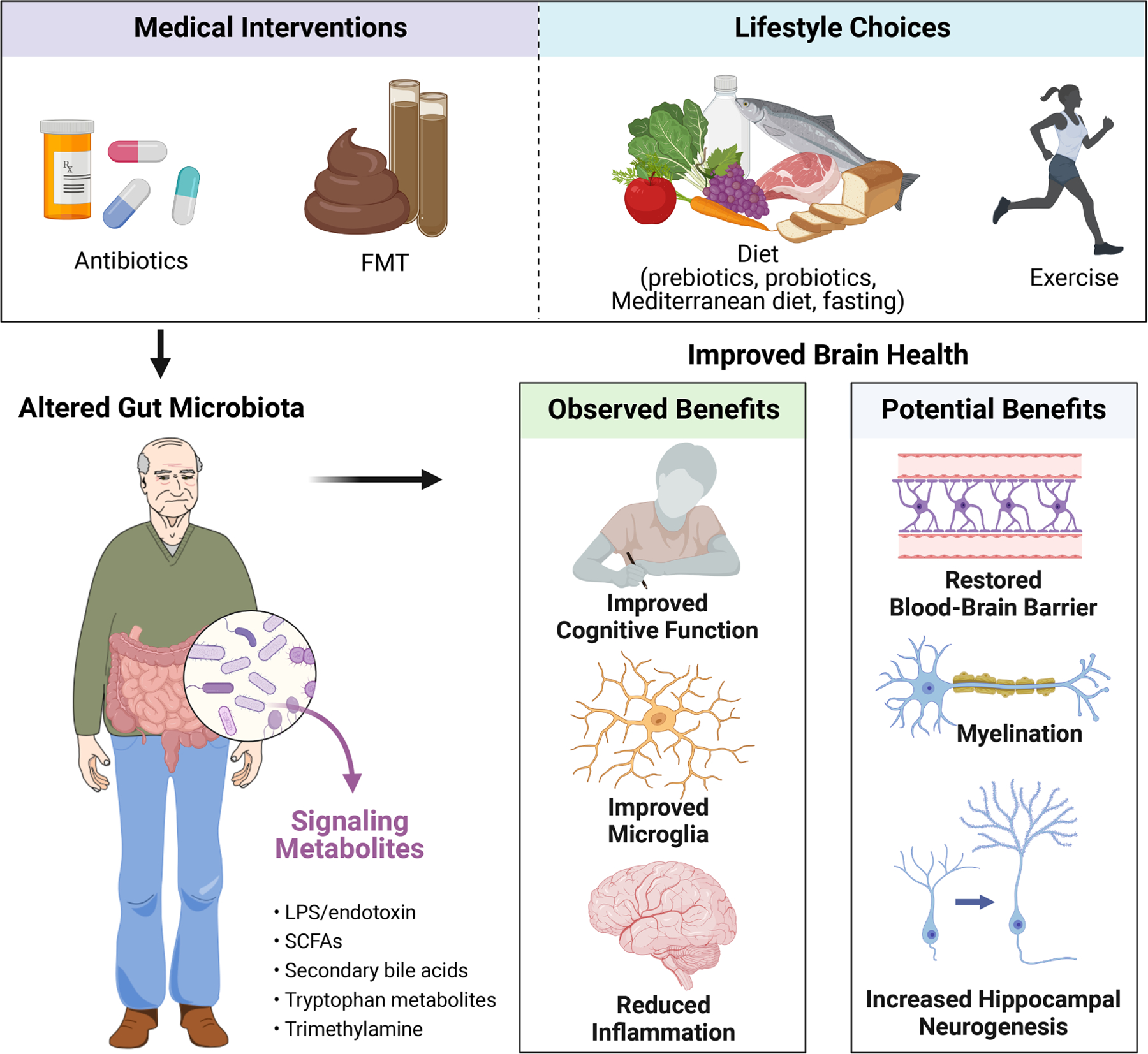
Potential strategies for intervening on the microbiota–gut–brain axis for improved brain health in ageing. Abbreviations: FMT, faecal microbiota transplant; LPS, lipopolysaccharides; SCFAs, short-chain fatty acids. Created with BioRender.com.

As the causal relationships between the gut microbiota and host brain ageing become increasingly clear, it is critical to continue to investigate whether microbiota-targeted therapeutics hold the potential to ameliorate the effects of ageing onto the brain. Several approaches to altering the gut microbiota, including medical interventions such as FMT and antibiotics, as well as lifestyle choices such as diet including probiotics, prebiotics, Mediterranean diet and intermittent fasting, and exercise, may hold the key to the fountain of brain youth (Figure 2). In the following section, we review the existing evidence with a focus on clinical evidence, accompanied with preclinical data as supporting evidence.

### Faecal microbiota transplant

Faecal microbiota transplant is now a commonly performed medical procedure to treat patients suffering from antibiotic-resistant *C. difficile*, and is being explored for use in a variety of other diseases and disorders, including pathologies impacting brain health (Sorbara and Pamer, 2022). Fascinatingly, smallscale clinical trials have shown that FMT can alleviate symptoms in patients with Parkinson’s disease (Kuai et al., 2021; Xue et al., 2020), although these studies are limited by small sample size. Furthermore, individual case studies have shown that FMT was sufficient to improve metrics of mobility in a patient with multiple sclerosis (Engen et al., 2020), and improve cognitive performance in two individuals diagnosed with Alzheimer’s disease who were administered FMT to treat their *C. difficile* infections (Hazan, 2020; Park et al., 2021). This evidence provides promise that FMT may offer a potential treatment option for patients suffering ageing-related neurological diseases.

Still, clinical FMT presents safety, logistical and feasibility questions, especially when operating within a community that has a higher incidence of frailty and immunodeficiency (Marquez et al., 2020). Therefore, although preclinical FMT studies have shown promise at improving brain health during ageing (Boehme, Guzzetta, Bastiaanssen et al., 2021; Mossad et al., 2021; Parker et al., 2022), it would be highly beneficial to develop alternative, less invasive and hazardous strategies to remodel the microbiome–gut–brain axis, which would allow physicians to move beyond faecal microbiota transplantation.

### Antibiotics

Antibiotics are commonly administered to treat infections and antibiotic usage by elderly populations is rising substantially, making it critical to understand whether there are off-target negative or beneficial consequences to antibiotic usage in ageing. For instance, antibiotics can have off-target implications by harming beneficial microbes or resulting in the emergence of antibiotic resistance (Barbosa and Levy, 2000). Alternatively, antibiotics that eradicate a harmful gut microbe may be able to improve brain health, though this is largely unexplored. Furthermore, several orally administered antibiotics can leak from the gut, allowing them to act directly on other systems including the brain regardless of the microbiota–gut–brain axis. This is of higher concern in disease states where barrier function is known to be compromised (Nau et al., 2010).

Compelling preclinical evidence demonstrates that antibiotics may be therapeutic for neurodegeneration when administered to rodent models of AD, as discussed earlier. While clinical evidence is largely lacking, one pilot, open-label trial involving 10 patients with mild to moderate probable AD dementia found that treatment with rifaximin (a minimally absorbed antibiotic) for 3 months significantly reduced neurofilament-light levels in the serum and modestly improved other serum markers for disease severity, although no improvements to cognition were observed in this timeframe (Suhocki et al., 2022). Interestingly, another study administering rifaximin to patients with cirrhosis and cognitive impairment found that rifaximin treatment improved working memory and inhibitory control, along with enhanced fronto-parietal and subcortical activation and connectivity (Ahluwalia et al., 2014).

Nonetheless, antibiotics may not be the best treatment approach for multiple reasons, including their potential for triggering harmful neurotoxic side effects. For instance, broad-spectrum antibiotics may deplete beneficial symbiotic gut bacteria, which may be playing critical support roles to the brain during ageing. Several case reports have linked the consumption of antibiotics and high plasma levels of antibiotics with the onset of delirium in the elderly (Moore and O’Keeffe, 1999; Schliamser et al., 1991). Furthermore, one recent observational study involving over 14,000 women self-reporting their antibiotic use found associations between chronic antibiotic use during midlife with slightly reduced cognitive scores after a follow-up period of roughly 7 years (Mehta et al., 2022), although the type of antibiotic and the reason for chronic antibiotic use were not known. These few clinical publications implicating antibiotic use and cognitive function are also confounded by the requirement of antibiotics to treat a specific health condition, rather than solely investigating their impact on brain health.

Since it is unclear whether the harms of antibiotic use outweigh the potential benefits in individuals that are not actively battling a bacterial infection, and because antibiotic use supports the development of antibiotic-resistant bacteria, the potential for antibiotics as a therapeutic for ageing-associated neurodegenerative diseases may be limited. Therefore, the development of novel antibiotics, such as engineered phages that could specifically eliminate individual identified pathogenic bacteria, could become valuable in treating conditions wherein specific pathogenic gut microbes are identified.

### Probiotics

It is increasingly recognised that microbiome-host modulation through probiotics is a promising avenue to improve cognitive function in human ageing (Eastwood et al., 2021). Probiotics are defined as live microorganisms that, when administered in adequate amounts, confer a health benefit on the host (Hill et al., 2014). Recent work by researchers in Japan has shown evidence that probiotics improve cognition in elderly with mild cognitive impairment. In a randomised, double-blind, placebo-controlled trial, a group from Japan showed that a 4-month intervention with a *Bifidobacterium breve* strain could be an effective approach for improving memory functions of subjects with MCI showing improvements in various cognitive domains such as immediate, delayed and visuospatial memory (Xiao et al., 2020). Previously the same group investigated the effects of this strain on cognitive function of older adults with a range of memory complaints, and found that, once the cohort was stratified, this probiotic induced beneficial effects only in a sub-population with more severe mild cognitive impairment (Kobayashi et al., 2019). Intriguingly, new work from the group suggests that it may have an impact on age-related brain atrophy (Asaoka et al., 2022). In a Korean study, 12 weeks supplementation with *Lactobacillus plantarum* fermented soybean significantly improved a cognitive composite score which was predominantly driven by an improvement in attention in MCI subjects (Hwang et al., 2019). Compared to these, the first evidence in a Caucasian population showed efficacy in improving a cognitive composite score in elderly with MCI, with another strain in the *Lactobacillus* genus, *Lactobacillus rhamnosus GG* (Sanborn et al., 2020). Although these results are interesting, the design of the study demands caution. For example, the researchers were not blinded to the randomisation of the groups. The beneficial effects of *L. rhamnosus GG* could be mediated through immunomodulatory pathways (Hibberd et al., 2014; Schultz et al., 2003), which have been linked to poor cognitive performance in elderly (Baune et al., 2008; Trollor et al., 2010). In a small Japanese cohort, (Ueda et al. (2021) observed a reduced relative abundance of *Faecalibacterium prausnitzii* in elderly with MCI compared to healthy individuals. By isolating two *F. prausnitzii* strains from the healthy elderly group, the authors improved cognitive impairments in mice concomitant with changes in genes related to oxidative stress and mitochondrial function (Ueda et al., 2021). Notably, one of the *F. prausnitzii* strains was given in a pasteurised form suggesting that beneficial effects are not only restricted to live bacteria, which directly exert effects on the host but could be also mediated through effects elicited by extracellular membrane components or could act immunomodulatory. Another next generation probiotic besides *F. prausnitzii*, *A. muciniphila*, showed a similar phenomenon in a small RCT in humans exerting its positive effects on some metabolic parameters predominantly in its pasteurised form (Depommier et al., 2019). While there is currently no direct clinical evidence that *Akkermansia* improves cognition in elderly people, the possibility that *A. muciniphila* influences cognition-linked metabolic factors, such as insulin sensitivity (Willmann et al., 2020), suggests that it may have the potential for improving cognitive health during ageing.

The efficacy of probiotics was also assessed in AD with mixed findings (Agahi et al., 2018; Akbari et al., 2016). An Iranian study using a multispecies mixture containing different stains and species of the genera *Lactobacillus* and *Bifidobacterium* for 12 weeks, did not find a positive effect on cognition in patients with severe AD (Agahi et al., 2018). It is plausible that probiotics may not exert positive effects on cognition when the damage is too advanced. Supplementation with a probiotic milk containing *Lactobacillus acidophilus*, *Lactobacillus casei*, *Bifidobacterium bifidum* and *Lactobacillus fermentum* another Iranian study showed improved insulin sensitivity, lower levels of the inflammatory marker CRP, and an improved cognitive score in the Mini-Mental State Examination (MMSE) after 12-weeks supplementation (Akbari et al., 2016). Furthermore, one study found that a probiotic mix containing *L. acidophilus*, *B. bifidum* and *Bifidobacterium longum* combined with selenium improved the MMSE score in patients with AD (Tamtaji et al., 2019), while another study in a Caucasian population did not find benefits on a cognitive score in AD with a multispecies probiotic consisting of *L. casei W56*, *L. lactis W19*, *L. acidophilus W22*, *B. lactis W52*, *L. paracasei W20*, *L. plantarum W62*, *B. lactis W51*, *B. bifidum W23* and *L. salivarius W24* (Leblhuber et al., 2018) possibly due to the shorter intervention time. The criteria for recruiting subjects across MCI and AD studies differ substantially. Similarly, the evaluation metrics for cognitive assessments also differ. Larger, well-controlled RCTs with harmonised inclusion and cognitive evaluation criteria, and longer interval follow-ups are needed to provide more reliable evidence (Xiang et al., 2022).

Apart from interventions with classical lactic acid bacteria, studies utilising preclinical models showed that a combination of five *Lactobacillus* strains with five Enterococcus strains had beneficial effects on high-fat diet–induced disturbances of the microbiome, intestinal permeability, metabolism, immunity ultimately improving physical decline in older mice (Ahmadi et al., 2020). Interestingly, a recent preclinical study suggests that *Enterococcus faecalis* plays a role in regulating social behaviour and social stress-induced stress response in mice (Wu et al., 2021b) though ageing was not considered in this paper.

Apart from studies examining older individuals with dementia or pre-dementia status, there is very limited evidence of the beneficial effects of probiotics on cognition in healthy older adults with memory complaints. Herein, probiotic-induced improvements have thus far been observed only under conditions where additional stressors were present (Eastwood et al., 2021). For instance, one Korea-based study found that *Lactobacillus helveticus* IDCC3801 fermented milk improved cognitive functioning during cognitive fatigue tests (Chung et al., 2014). It seems that the cognitive status and accompanied underlying physiological changes could be predictive for the susceptibility of an individual to cognitive decline, although more studies are needed to understand the factors driving sensitivity to probiotic interventions.

Studies on the use of probiotics for PD are sparse. Pilot data in an open-label, single-arm, baseline-controlled trial suggests possible benefits on motor skills and quality of life with *L. plantarum* PS128 (Lu et al., 2021). Further placebo-controlled studies are needed to support these initial results.

Certain clinical studies are looking into the prevention of neurodegenerative conditions through microbiome-targeted strategies utilising microbial metabolites such as SCFAs. Duscha et al. (2020) have shown that two weeks of propionic acid intake leads to a sustained increase of immunoregulatory T (Treg) cells while three years of propionic acid intake positively influenced functional parameters through a reduced relapse rate associated with reduced brain atrophy. In addition, preclinical models have shown that modulation of neuroinflammation and cognitive decline can be achieved through oral supplementation with butyrate (Govindarajan et al., 2011; Matt et al., 2018), however, these findings have yet to be explored in humans. In contrast, a population-based cohort in France with over 9,000 community dwellers aged 65 years or more, found that increased propionic acid in serum was associated with increased odds of cognitive decline (Neuffer et al., 2022). Mediation analyses suggested that this adverse association may be mediated through hypercholesterolemia and glycaemia with a strong correlation with a fasting blood glucose suggesting metabolic disruption as a possible pathway in relation of propionic acid to cognitive health (Neuffer et al., 2022). Overall these data suggests that the effect of microbial metabolites are context-dependent where further research is needed to understand how underlying conditions can prevent or perturbate the effects of microbial metabolites.

Intriguingly, a recent study suggests that the bacteriophages group *Caudovirales* can improve executive function and memory in both preclinical models and in humans (Mayneris-Perxachs et al., 2022). However, the impact of bacteriophages on cognitive health in the elderly remains underexplored. It is plausible that some of the apparent effects that bacteria have on the host may be in part mediated or influenced by bacteriophages (Teng et al., 2022).

### Prebiotics

Clinical evidence for prebiotics, substrates that are selectively utilised by host microorganisms conferring a health benefit (Gibson et al., 2017), to improve brain health in ageing is limited. Despite this lack of research, there is some evidence that a prebiotic mix containing inulin and fructo-oligosaccharides can improve frailty, a risk factor for cognitive decline (Borges et al., 2019), under certain conditions (Buigues et al., 2016; Theou et al., 2019) without showing a benefit on cognition (Buigues et al., 2016). Preclinically, supplementation with inulin decreased neuroinflammation in brains of middle-aged mice (Boehme et al., 2020), while inulin in combination with *Enterococcus faecium* (Romo-Araiza et al., 2018) showed similar effects in aged mice (Matt et al., 2018), and modulated brain ageing-related metabolites such as TMAO following chronic stress in the brains of aged mice (Cruz-Pereira et al., 2022). Possible mechanisms may include microbial metabolite signalling-mediated modulation of peripheral and brain immune activation, modulation of brain metabolites, stimulation of gut hormones and neurotransmitter signalling (Boehme et al., 2020; Matt et al., 2018; Stilling et al., 2016; van de Wouw et al., 2019). Moreover, the combination of the prebiotic fructo-oligosaccharide with multiple bacterial strains (*L. paracasei*, *L. rhamnosus*, *L. acidophilus* and *B. lactis*) mildly enhanced cognitive performance in healthy elderly (Louzada and Ribeiro, 2020).

### Diet

Novel dietary interventions modulating the gut microbiota composition has been proposed to combat age-related decline and improve physiological well-being (Cryan et al., 2019a). From epidemiological studies, compelling evidence suggests that Mediterranean diet can be beneficial for the ageing human brain (van de Rest et al., 2015). Components of a Mediterranean diet such as Omega-3 polyunsaturated fatty acids (O3-PUFAs) or vitamin A have been shown to elicit protective effects on the ageing brain in preclinical models (Labrousse et al., 2012; Touyarot et al., 2013). Also, polyphenols have been shown to positively influence cognition in preclinical and clinical settings (de Vries et al, 2021). Conversely, these nutrients have been shown to modulate the gut microbiota and can rescue microbiome-associated perturbations on brain health in preclinical models (Donoso et al., 2020; Provensi et al., 2019). Work from the NU-AGE consortium shed light onto the relationship between Mediterranean diet-induced changes in the gut microbiota and their link to improved health in elderly, showing reduced frailty and improved health status associated with an intermediate microbiome response (Ghosh et al., 2020). Adherence to the Mediterranean diet was linked to increased abundance in taxa which were positively associated with improved health outcomes such as decreased frailty and improved cognition, and negatively associated with inflammatory markers such as C-reactive protein and interleukin-17 (IL-17) suggesting that Mediterranean diet may have the potential to promote healthier ageing (Ghosh et al., 2020). Accumulating preclinical and clinical research highlights IL-17 as a major cytokine involved in neurodegenerative processes and cognitive decline (Brigas et al., 2021; Cipollini et al., 2019; Regen et al., 2021), suggesting that dietary strategies which decrease IL-17 could promote healthy ageing (Dupraz et al., 2021). Notably, elderly individuals who maintained high adherence showed improved global cognition and episodic memory after one year compared to low adherence (Marseglia et al., 2018) which were associated with alterations in their gut microbiome, suggesting that broad dietary intervention could be considered as a less invasive strategy than FMT for altering the gut microbiota towards improved cognition in elderly people (Ghosh et al., 2020). Analysis of inferred microbial functions showed an increase in short and branch chained fatty acid production and lower production of secondary bile acids (Ghosh et al., 2020). Of note, metabolic network analysis in clinical studies of AD points to a possible link of secondary bile acids with deterioration of cognition (Baloni et al., 2020). In a large multicentre study, the authors report here an association between altered bile acid profile, genetic variants implicated in Alzheimer’s disease, and cognitive changes (MahmoudianDehkordi et al., 2019). Adherence to the Mediterranean diet has further shown to slow down the progression of AD (Berti et al., 2018). The impact on dementia-type cognitive impairment has been also assessed by introducing a broader change in diet, which holds promise towards a more profound adjustment of the gut microbiome as opposed to the use of supplements (Del Bo et al., 2021; Ghosh et al., 2022a,b; Nagpal et al., 2019). In a pilot study using a modified Mediterranean-ketogenic diet, the authors found changes in several taxa with distinct patterns between cognitively impaired versus cognitively normal subjects (Nagpal et al., 2019).

Vice versa, western-style diets, rich in saturated fat and high in sugar, may trigger cognitive dysfunction in ageing (Beilharz et al., 2015; Gardener et al., 2015; Wieckowska-Gacek et al., 2021). A recent study shed more light into the role of the microbiota in this process showing a microbiota-dependent accumulation of the advanced glycation end product N6-carboxymethyllysine in microglia of aged mice linked to oxidative stress and mitochondrial dysfunction (Mossad et al., 2022), risk factors for cognitive impairment. Furthermore, calorie restriction resulted in a lower level of amyloid plaque in a mouse model of AD (Cox et al., 2019). Intermittent fasting and caloric restriction emerged as a viable strategy to improve cognition not only in mouse models of ageing and Alzheimer’s disease and primates, but also in humans (Chakraborty et al., 2020; Dal-Pan et al., 2011; Ooi et al., 2020; Pan et al., 2022; Witte et al., 2009). While the level and feasibility of caloric restriction in humans remains to be elucidated, intermittent fasting represents a possible strategy to improve cognitive health during ageing.

## Conclusions and future perspectives

Research published over the last few years has demonstrated that the gut microbiota is a crucial contributor to (and potential target to improve) host brain ageing, including in neurodegenerative diseases, making this an exciting time for research within this scope. Indeed, microbiota-targeted interventions have shown promise for improving cognitive performance and overall brain health during ageing. While this field of research is very young and mechanistic evidence is entirely preclinical, clinical studies involving probiotics and dietary interventions that alter the gut microbiota have shown success at improving aspects of cognition in older people. Furthermore, clinical trials and case studies involving FMT demonstrate promise for improving symptoms of neurodegenerative diseases, including PD, AD and MS, giving hope to the future of medicine for the thousands of individuals living with these diseases who currently have limited treatment options. Nonetheless, this is a rapidly emerging field, and large-scale, well-controlled clinical studies are required to better characterise the potential for the gut microbiota as a treatment target within these diseases.

Ageing is a complex biological process, and individual’s age at different speeds and trajectories, which complicates defining healthy ageing, including how the gut microbiota should be modulated to support healthy ageing. Moreover, the impact of biological sex on ageing remains understudied, including how sex shapes age-related physiological changes in the microbiota-host dialogue, regardless of the increasing evidence that suggests biological sex contributes to the susceptibility for several age-related diseases. While the gut microbiota may become a diagnostic tool to better understand an individual’s biological age(ing) (Ratiner et al., 2022), it may be necessary to tailor a microbiota-targeted intervention to an individual’s ageing process, considering individualised factors such as lifestyle, dietary habits and its existing resident gut microbiome. Therefore, there may not be a one-size-fits-all solution to improving brain ageing via the gut microbiota, and instead personalised solutions may be required. Increasing our understanding on ecological aspects in diet-microbiome interrelationship and its impact on the host are key going forward in designing efficacious nutritional interventions. This also includes an understanding on how the resident gut microbiome may impact the efficacy of nutritional solutions on host outcomes (Maldonado-Gomez et al., 2016; Shepherd et al., 2018), taking into account factors such as geographical, genetic and lifestyle differences across populations (Deschasaux et al., 2018).

A dominant, lingering question is the definition of a healthy gut microbiome, and whether a universal microbiome indicative of health during ageing indeed exists (Hill, 2020; Shanahan et al., 2021). Technological advances including high-resolution next-generation sequencing, metabolomics, transcriptomics, proteomics and machine learning and our knowledge of microbial ecology will drive forward our understanding of the microbiota–gut–brain axis in healthy brain ageing. Further well-designed randomised-controlled clinical studies, mechanistic investigations and large-scale population studies must be conducted to better understand the role of microbiota-targeted therapeutics for brain health during ageing. Elucidating the factors which drive individualised responses and outcomes are key to designing personalised microbiome-targeted interventions to improve physiology and brain function in ageing.

## References

[R1] Agahi A, Hamidi GA, Daneshvar R, Hamdieh M, Soheili M, Alinaghipour A, Esmaeili Taba SM and Salami M (2018) Does severity of Alzheimer’s disease contribute to its responsiveness to modifying gut microbiota? A double blind clinical trial. Frontiers in Neurology 9, 662. 10.3389/fneur.2018.00662 30158897 PMC6104449

[R2] Ahluwalia V, Wade JB, Heuman DM, Hammeke TA, Sanyal AJ, Sterling RK, Stravitz RT, Luketic V, Siddiqui MS, Puri P, Fuchs M, Lennon MJ, Kraft KA, Gilles H, White MB, Noble NA and Bajaj JS (2014) Enhancement of functional connectivity, working memory and inhibitory control on multi-modal brain MR imaging with rifaximin in cirrhosis: Implications for the gut-liver-brain axis. Metabolic Brain Disease 29(4), 1017–1025. 10.1007/s11011-014-9507-6 24590688 PMC4155029

[R3] Ahmadi S, Wang S, Nagpal R, Wang B, Jain S, Razazan A, Mishra SP, Zhu X, Wang Z, Kavanagh K and Yadav H (2020) A human-origin probiotic cocktail ameliorates aging-related leaky gut and inflammation via modulating the microbiota/taurine/tight junction axis. JCI Insight 5(9), e132055. 10.1172/jci.insight.132055 32302292 PMC7253024

[R4] Akbari E, Asemi Z, Daneshvar Kakhaki R, Bahmani F, Kouchaki E, Tamtaji OR, Hamidi GA and Salami M (2016) Effect of probiotic supplementation on cognitive function and metabolic status in Alzheimer’s disease: A randomized, double-blind and controlled trial. Frontiers in Aging Neuroscience 8, 256. 10.3389/fnagi.2016.00256 27891089 PMC5105117

[R5] Alkasir R, Li J, Li X, Jin M and Zhu B (2017) Human gut microbiota: The links with dementia development. Protein & Cell 8(2), 90–102. 10.1007/s13238-016-0338-6 27866330 PMC5291774

[R6] Ambrosini YM, Borcherding D, Kanthasamy A, Kim HJ, Willette AA, Jergens A, Allenspach K and Mochel JP (2019) The gut-brain axis in neurodegenerative diseases and relevance of the canine model: A review. Frontiers in Aging Neuroscience 11, 130. 10.3389/fnagi.2019.00130 31275138 PMC6591269

[R7] Asaoka D, Xiao J, Takeda T, Yanagisawa N, Yamazaki T, Matsubara Y, Sugiyama H, Endo N, Higa M, Kasanuki K, Ichimiya Y, Koido S, Ohno K, Bernier F, Katsumata N, Nagahara A, Arai H, Ohkusa T and Sato N (2022) Effect of probiotic Bifidobacterium breve in improving cognitive function and preventing brain atrophy in older patients with suspected mild cognitive impairment: Results of a 24-week randomized, double-blind placebo-controlled trial. Journal of Alzheimers Diseases 88(1), 75–95. 10.3233/JAD-220148 PMC9277669 35570493

[R8] Badal VD, Vaccariello ED, Murray ER, Yu KE, Knight R, Jeste DV and Nguyen TT (2020) The gut microbiome, aging, and longevity: A systematic review. Nutrients 12(12), 3759. 10.3390/nu12123759 33297486 PMC7762384

[R9] Baloni P, Funk CC, Yan J, Yurkovich JT, Kueider-Paisley A, Nho K, Heinken A, Jia W, Mahmoudiandehkordi S, Louie G, Saykin AJ, Arnold M, Kastenmuller G, Griffiths WJ, Thiele I, Alzheimer’s Disease Metabolomics C, Kaddurah-Daouk R and Price ND (2020) Metabolic network analysis reveals altered bile acid synthesis and metabolism in Alzheimer’s disease. Cell Reports Medicine 1(8), 100138. 10.1016/j.xcrm.2020.100138 33294859 PMC7691449

[R10] Barbosa TM and Levy SB (2000) The impact of antibiotic use on resistance development and persistence. Drug Resistance Updates 3(5), 303–311. 10.1054/drup.2000.0167 11498398

[R11] Barcena C, Valdes-Mas R, Mayoral P, Garabaya C, Durand S, Rodriguez F, Fernandez-Garcia MT, Salazar N, Nogacka AM, Garatachea N, Bossut N, Aprahamian F, Lucia A, Kroemer G, Freije JMP, Quiros PM and Lopez-Otin C (2019) Healthspan and lifespan extension by fecal microbiota transplantation into progeroid mice. Nature Medicine 25(8), 1234–1242. 10.1038/s41591-019-0504-5 31332389

[R12] Baune BT, Ponath G, Golledge J, Varga G, Arolt V, Rothermundt M and Berger K (2008) Association between IL-8 cytokine and cognitive performance in an elderly general population – The MEMO-study. Neurobiology of Aging 29(6), 937–944. 10.1016/j.neurobiolaging.2006.12.003 17207897

[R13] Beilharz JE, Maniam J and Morris MJ (2015) Diet-induced cognitive deficits: The role of fat and sugar, potential mechanisms and nutritional interventions. Nutrients 7(8), 6719–6738. 10.3390/nu7085307 26274972 PMC4555146

[R14] Berer K, Gerdes LA, Cekanaviciute E, Jia X, Xiao L, Xia Z, Liu C, Klotz L, Stauffer U, Baranzini SE, Kumpfel T, Hohlfeld R, Krishnamoorthy G and Wekerle H (2017) Gut microbiota from multiple sclerosis patients enables spontaneous autoimmune encephalomyelitis in mice. Proceedings of the National Academy of Sciences of the United States of America 114(40), 10719–10724. 10.1073/pnas.1711233114 28893994 PMC5635914

[R15] Berti V, Walters M, Sterling J, Quinn CG, Logue M, Andrews R, Matthews DC, Osorio RS, Pupi A, Vallabhajosula S, Isaacson RS, de Leon MJ and Mosconi L (2018) Mediterranean diet and 3-year Alzheimer brain biomarker changes in middle-aged adults. Neurology 90(20), e1789–e1798. 10.1212/WNL.0000000000005527 29653991 PMC5957301

[R16] Biagi E, Franceschi C, Rampelli S, Severgnini M, Ostan R, Turroni S, Consolandi C, Quercia S, Scurti M, Monti D, Capri M, Brigidi P and Candela M (2016) Gut microbiota and extreme longevity. Current Biology 26(11), 1480–1485. 10.1016/j.cub.2016.04.016 27185560

[R17] Biagi E, Nylund L, Candela M, Ostan R, Bucci L, Pini E, Nikkila J, Monti D, Satokari R, Franceschi C, Brigidi P and De Vos W (2010) Through ageing, and beyond: Gut microbiota and inflammatory status in seniors and centenarians. PLoS One 5(5), e10667. 10.1371/journal.pone.0010667 20498852 PMC2871786

[R18] Biagi E, Rampelli S, Turroni S, Quercia S, Candela M and Brigidi P (2017) The gut microbiota of centenarians: Signatures of longevity in the gut microbiota profile. Mechanisms of Ageing and Development 165(Pt B), 180–184. 10.1016/j.mad.2016.12.013 28049008

[R19] Blacher E, Bashiardes S, Shapiro H, Rothschild D, Mor U, Dori-Bachash M, Kleimeyer C, Moresi C, Harnik Y, Zur M, Zabari M, Brik RB, Kviatcovsky D, Zmora N, Cohen Y, Bar N, Levi I, Amar N, Mehlman T, Brandis A, Biton I, Kuperman Y, Tsoory M, Alfahel L, Harmelin A, Schwartz M, Israelson A, Arike L, Johansson MEV, Hansson GC, Gotkine M, Segal E and Elinav E (2019) Potential roles of gut microbiome and metabolites in modulating ALS in mice. Nature 572(7770), 474–480. 10.1038/s41586-019-1443-5 31330533

[R20] Boehme M, Guzzetta KE, Bastiaanssen TFS, van de Wouw M, Moloney GM, Gual-Grau A, Spichak S, Olavarría-Ramírez L, Fitzgerald P, Morillas E, Ritz NL, Jaggar M, Cowan CSM, Crispie F, Donoso F, Halitzki E, Neto MC, Sichetti M, Golubeva AV, Fitzgerald RS, Claesson MJ, Cotter PD, O’Leary OF, Dinan TG and Cryan JF (2021) Microbiota from young mice counteracts selective age-associated behavioral deficits. Nature Aging 1(8), 666–676. 10.1038/s43587-021-00093-9 37117767

[R21] Boehme M, van de Wouw M, Bastiaanssen TFS, Olavarría-Ramírez L, Lyons K, Fouhy F, Golubeva AV, Moloney GM, Minuto C, Sandhu KV, Scott KA, Clarke G, Stanton C, Dinan TG, Schellekens H and Cryan JF (2020) Mid-life microbiota crises: Middle age is associated with pervasive neuroimmune alterations that are reversed by targeting the gut microbiome. Molecular Psychiatry 25(10), 2567–2583. 10.1038/s41380-019-0425-1 31092898

[R22] Borges MK, Canevelli M, Cesari M and Aprahamian I (2019) Frailty as a predictor of cognitive disorders: A systematic review and meta-analysis. Frontiers in Medicine (Lausanne) 6, 26. 10.3389/fmed.2019.00026 PMC6389599 30838210

[R23] Bosco N and Noti M (2021) The aging gut microbiome and its impact on host immunity. Genes and Immunity 22(5–6), 289–303. 10.1038/s41435-021-00126-8 33875817 PMC8054695

[R24] Braniste V, Al-Asmakh M, Kowal C, Anuar F, Abbaspour A, Toth M, Korecka A, Bakocevic N, Ng LG, Kundu P, Gulyas B, Halldin C, Hultenby K, Nilsson H, Hebert H, Volpe BT, Diamond B and Pettersson S (2014) The gut microbiota influences blood–brain barrier permeability in mice. Science Translational Medicine 6(263), 263ra158. 10.1126/scitranslmed.3009759 PMC4396848 25411471

[R25] Bretherton B, Atkinson L, Murray A, Clancy J, Deuchars S and Deuchars J (2019) Effects of transcutaneous vagus nerve stimulation in individuals aged 55 years or above: Potential benefits of daily stimulation. Aging (Albany NY) 11(14), 4836–4857. 10.18632/aging.102074 31358702 PMC6682519

[R26] Brigas HC, Ribeiro M, Coelho JE, Gomes R, Gomez-Murcia V, Carvalho K, Faivre E, Costa-Pereira S, Darrigues J, de Almeida AA, Buee L, Dunot J, Marie H, Pousinha PA, Blum D, Silva-Santos B, Lopes LV and Ribot JC (2021) IL-17 triggers the onset of cognitive and synaptic deficits in early stages of Alzheimer’s disease. Cell Reports 36(9), 109574. 10.1016/j.celrep.2021.109574 34469732

[R27] Buigues C, Fernandez-Garrido J, Pruimboom L, Hoogland AJ, Navarro-Martinez R, Martinez-Martinez M, Verdejo Y, Mascaros MC, Peris C and Cauli O (2016) Effect of a prebiotic formulation on frailty syndrome: A randomized, double-blind clinical trial. International Journal of Molecular Sciences 17(6). 10.3390/ijms17060932 PMC4926465 27314331

[R28] Cahill LS, Zhang MA, Ramaglia V, Whetstone H, Sabbagh MP, Yi TJ, Woo L, Przybycien TS, Moshkova M, Zhao FL, Rojas OL, Gomes J, Kuerten S, Gommerman JL, Sled JG and Dunn SE (2019) Aged hind-limb clasping experimental autoimmune encephalomyelitis models aspects of the neurodegenerative process seen in multiple sclerosis. Proceedings of the National Academy of Sciences of the United States of America 116(45), 22710–22720. 10.1073/pnas.1915141116 31641069 PMC6842635

[R29] Cekanaviciute E, Yoo BB, Runia TF, Debelius JW, Singh S, Nelson CA, Kanner R, Bencosme Y, Lee YK, Hauser SL, Crabtree-Hartman E, Sand IK, Gacias M, Zhu Y, Casaccia P, Cree BAC, Knight R, Mazmanian SK and Baranzini SE (2017) Gut bacteria from multiple sclerosis patients modulate human T cells and exacerbate symptoms in mouse models. Proceedings of the National Academy of Sciences of the United States of America 114(40), 10713–10718. 10.1073/pnas.1711235114 28893978 PMC5635915

[R30] Cereda E, Sansone V, Meola G and Malavazos AE (2007) Increased visceral adipose tissue rather than BMI as a risk factor for dementia. Age and Ageing 36(5), 488–491. 10.1093/ageing/afm096 17656423

[R31] Chakraborty A, Banerjee S, Mukherjee B and Poddar MK (2020) Calorie restriction improves aging-induced impairment of cognitive function in relation to deregulation of corticosterone status and brain regional GABA system. Mechanisms of Ageing and Development 189, 111248. 10.1016/j.mad.2020.111248 32339520

[R32] Chu H and Mazmanian SK (2013) Innate immune recognition of the microbiota promotes host-microbial symbiosis. Nature Immunology 14(7), 668–675. 10.1038/ni.2635 23778794 PMC4109969

[R33] Chung Y-C, Jin H-M, Cui Y, Kim DS, Jung JM, Park J-I, Jung E-S, Choi E-K and Chae S-W (2014) Fermented milk of *Lactobacillus helveticus* IDCC3801 improves cognitive functioning during cognitive fatigue tests in healthy older adults. Journal of Functional Foods 10, 465–474. 10.1016/j.jff.2014.07.007

[R34] Cipollini V, Anrather J, Orzi F and Iadecola C (2019) Th17 and cognitive impairment: Possible mechanisms of action. Frontiers in Neuroanatomy 13, 95. 10.3389/fnana.2019.00095 31803028 PMC6877481

[R35] Claesson MJ, Cusack S, O’Sullivan O, Greene-Diniz R, de Weerd H, Flannery E, Marchesi JR, Falush D, Dinan T, Fitzgerald G, Stanton C, van Sinderen D, O’Connor M, Harnedy N, O’Connor K, Henry C, O’Mahony D, Fitzgerald AP, Shanahan F, Twomey C, Hill C, Ross RP and O’Toole PW (2011) Composition, variability, and temporal stability of the intestinal microbiota of the elderly. Proceedings of the National Academy of Sciences of the United States of America 108(Suppl 1), 4586–4591. 10.1073/pnas.1000097107 20571116 PMC3063589

[R36] Claesson MJ, Jeffery IB, Conde S, Power SE, O’Connor EM, Cusack S, Harris HM, Coakley M, Lakshminarayanan B, O’Sullivan O, Fitzgerald GF, Deane J, O’Connor M, Harnedy N, O’Connor K, O’Mahony D, van Sinderen D, Wallace M, Brennan L, Stanton C, Marchesi JR, Fitzgerald AP, Shanahan F, Hill C, Ross RP and O’Toole PW (2012) Gut microbiota composition correlates with diet and health in the elderly. Nature 488(7410), 178–184. 10.1038/nature11319 22797518

[R37] Cox LM, Maghzi AH, Liu S, Tankou SK, Dhang FH, Willocq V, Song A, Wasen C, Tauhid S, Chu R, Anderson MC, De Jager PL, Polgar-Turcsanyi M, Healy BC, Glanz BI, Bakshi R, Chitnis T and Weiner HL (2021) Gut microbiome in progressive multiple sclerosis. Annals of Neurology 89(6), 1195–1211. 10.1002/ana.26084 33876477 PMC8132291

[R38] Cox LM, Schafer MJ, Sohn J, Vincentini J, Weiner HL, Ginsberg SD and Blaser MJ (2019) Calorie restriction slows age-related microbiota changes in an Alzheimer’s disease model in female mice. Scientific Reports 9(1), 17904. 10.1038/s41598-019-54187-x 31784610 PMC6884494

[R39] Cruz-Pereira JS, Moloney GM, Bastiaanssen TFS, Boscaini S, Tofani G, Borras-Bisa J, van de Wouw M, Fitzgerald P, Dinan TG, Clarke G and Cryan JF (2022) Prebiotic supplementation modulates selective effects of stress on behavior and brain metabolome in aged mice. Neurobiology of Stress 21, 100501. 10.1016/j.ynstr.2022.100501 36532371 PMC9755060

[R40] Cryan JF, Boehme M and Dinan TG (2019a) Is the fountain of youth in the gut microbiome? The Journal of Physiology 597(9),2323–2324. 10.1113/JP277784 30875099 PMC6487933

[R41] Cryan JF, O’Riordan KJ, Cowan CSM, Sandhu KV, Bastiaanssen TFS, Boehme M, Codagnone MG, Cussotto S, Fulling C, Golubeva AV, Guzzetta KE, Jaggar M, Long-Smith CM, Lyte JM, Martin JA, Molinero-Perez A, Moloney G, Morelli E, Morillas E, O’Connor R, Cruz-Pereira JS, Peterson VL, Rea K, Ritz NL, Sherwin E, Spichak S, Teichman EM, van de Wouw M, Ventura-Silva AP, Wallace-Fitzsimons SE, Hyland N, Clarke G and Dinan TG (2019b) The microbiota-gut-brain axis. Physiological Reviews 99(4), 1877–2013. 10.1152/physrev.00018.2018 31460832

[R42] D’Amato A, Di Cesare Mannelli L, Lucarini E, Man AL, Le Gall G, Branca JJV, Ghelardini C, Amedei A, Bertelli E, Regoli M, Pacini A, Luciani G, Gallina P, Altera A, Narbad A, Gulisano M, Hoyles L, Vauzour D and Nicoletti C (2020) Faecal microbiota transplant from aged donor mice affects spatial learning and memory via modulating hippocampal synaptic plasticity- and neurotransmission-related proteins in young recipients. Microbiome 8(1), 140. 10.1186/s40168-020-00914-w 33004079 PMC7532115

[R43] Dal-Pan A, Pifferi F, Marchal J, Picq JL, Aujard F and Consortium R (2011) Cognitive performances are selectively enhanced during chronic caloric restriction or resveratrol supplementation in a primate. PLoS One 6(1), e16581. 10.1371/journal.pone.0016581 21304942 PMC3031601

[R44] de Vries K, Medawar E, Korosi A and Witte AV (2021) The effect of polyphenols on working and episodic memory in nonpathological and pathological aging: A systematic review and meta-analysis. Frontiers in Nutrition 8, 720756. 10.3389/fnut.2021.720756 35155509 PMC8826433

[R45] Del Bo C, Bernardi S, Cherubini A, Porrini M, Gargari G, Hidalgo-Liberona N, Gonzalez-Dominguez R, Zamora-Ros R, Peron G, Marino M, Gigliotti L, Winterbone MS, Kirkup B, Kroon PA, Andres-Lacueva C, Guglielmetti S and Riso P (2021) A polyphenol-rich dietary pattern improves intestinal permeability, evaluated as serum zonulin levels, in older subjects: The MaPLE randomised controlled trial. Clinical Nutrition 40(5), 3006–3018. 10.1016/j.clnu.2020.12.014 33388204

[R46] Depommier C, Everard A, Druart C, Plovier H, Van Hul M, Vieira-Silva S, Falony G, Raes J, Maiter D, Delzenne NM, de Barsy M, Loumaye A, Hermans MP, Thissen JP, de Vos WM and Cani PD (2019) Supplementation with *Akkermansia muciniphila* in overweight and obese human volunteers: A proof-of-concept exploratory study. Nature Medicine 25(7), 1096–1103. 10.1038/s41591-019-0495-2 PMC6699990 31263284

[R47] Deschasaux M, Bouter KE, Prodan A, Levin E, Groen AK, Herrema H, Tremaroli V, Bakker GJ, Attaye I, Pinto-Sietsma S-J, van Raalte DH, Snijder MB, Nicolaou M, Peters R, Zwinderman AH, Bäckhed F and Nieuwdorp M (2018) Depicting the composition of gut microbiota in a population with varied ethnic origins but shared geography. Nature Medicine 24(10), 1526–1531. 10.1038/s41591-018-0160-1 30150717

[R48] Dinić M, Herholz M, Kačarević U, Radojević D, Novović K, Đokić J, Trifunović A and Golić N (2021) Host-commensal interaction promotes health and lifespan in *Caenorhabditis elegans* through the activation of HLH-30/TFEB-mediated autophagy. Aging (Albany NY) 13(6), 8040–8054. 10.18632/aging.202885 33770762 PMC8034897

[R49] Dobson R and Giovannoni G (2019) Multiple sclerosis – A review. European Journal of Neurology 26(1), 27–40. 10.1111/ene.13819 30300457

[R50] Dodiya HB, Frith M, Sidebottom A, Cao Y, Koval J, Chang E and Sisodia SS (2020) Synergistic depletion of gut microbial consortia, but not individual antibiotics, reduces amyloidosis in APPPS1–21 Alzheimer’s transgenic mice. Scientific Reports 10(1), 8183. 10.1038/s41598-020-64797-5 32424118 PMC7235236

[R51] Dodiya HB, Lutz HL, Weigle IQ, Patel P, Michalkiewicz J, Roman-Santiago CJ, Zhang CM, Liang Y, Srinath A, Zhang X, Xia J, Olszewski M, Zhang X, Schipma MJ, Chang EB, Tanzi RE, Gilbert JA and Sisodia SS (2021) Gut microbiota–driven brain Aβ amyloidosis in mice requires microglia. Journal of Experimental Medicine 219(1), e20200895. 10.1084/jem.20200895 34854884 PMC8647415

[R52] Dominy SS, Lynch C, Ermini F, Benedyk M, Marczyk A, Konradi A, Nguyen M, Haditsch U, Raha D, Griffin C, Holsinger LJ, Arastu-Kapur S, Kaba S, Lee A, Ryder MI, Potempa B, Mydel P, Hellvard A, Adamowicz K, Hasturk H, Walker GD, Reynolds EC, Faull RLM, Curtis MA, Dragunow M and Potempa J (2019) *Porphyromonas gingivalis* in Alzheimer’s disease brains: Evidence for disease causation and treatment with small-molecule inhibitors. Science Advances 5(1), eaau3333. 10.1126/sciadv.aau3333 30746447 PMC6357742

[R53] Donoso F, Egerton S, Bastiaanssen TFS, Fitzgerald P, Gite S, Fouhy F, Ross RP, Stanton C, Dinan TG and Cryan JF (2020) Polyphenols selectively reverse early-life stress-induced behavioural, neurochemical and microbiota changes in the rat. Psychoneuroendocrinology 116, 104673. 10.1016/j.psyneuen.2020.104673 32334345

[R54] Dupraz L, Magniez A, Rolhion N, Richard ML, Da Costa G, Touch S, Mayeur C, Planchais J, Agus A, Danne C, Michaudel C, Spatz M, Trottein F, Langella P, Sokol H and Michel ML (2021) Gut microbiota-derived short-chain fatty acids regulate IL-17 production by mouse and human intestinal γδ T cells. Cell Reports 36(1), 109332. 10.1016/j.celrep.2021.109332 34233192

[R55] Duscha A, Gisevius B, Hirschberg S, Yissachar N, Stangl GI, Eilers E, Bader V, Haase S, Kaisler J, David C, Schneider R, Troisi R, Zent D, Hegelmaier T, Dokalis N, Gerstein S, Del Mare-Roumani S, Amidror S, Staszewski O, Poschmann G, Stuhler K, Hirche F, Balogh A, Kempa S, Trager P, Zaiss MM, Holm JB, Massa MG, Nielsen HB, Faissner A, Lukas C, Gatermann SG, Scholz M, Przuntek H, Prinz M, Forslund SK, Winklhofer KF, Muller DN, Linker RA, Gold R and Haghikia A (2020) Propionic acid shapes the multiple sclerosis disease course by an immunomodulatory mechanism. Cell, 180(6), 1067–1080.e16. 10.1016/j.cell.2020.02.035 32160527

[R56] Eastwood J, Walton G, Van Hemert S, Williams C and Lamport D (2021) The effect of probiotics on cognitive function across the human lifespan: A systematic review. Neuroscience and Biobehavioral Reviews 128, 311–327. 10.1016/j.neubiorev.2021.06.032 34171323

[R57] Elliott ML, Caspi A, Houts RM, Ambler A, Broadbent JM, Hancox RJ, Harrington H, Hogan S, Keenan R, Knodt A, Leung JH, Melzer TR, Purdy SC, Ramrakha S, Richmond-Rakerd LS, Righarts A, Sugden K, Thomson WM, Thorne PR, Williams BS, Wilson G, Hariri AR, Poulton R and Moffitt TE (2021) Disparities in the pace of biological aging among midlife adults of the same chronological age have implications for future frailty risk and policy. Nature Aging 1(3), 295–308. 10.1038/s43587-021-00044-4 33796868 PMC8009092

[R58] Engen PA, Zaferiou A, Rasmussen H, Naqib A, Green SJ, Fogg LF, Forsyth CB, Raeisi S, Hamaker B and Keshavarzian A (2020) Single-arm, non-randomized, time series, single-subject study of fecal microbiota transplantation in multiple sclerosis. Frontiers in Neurology 11, 978. 10.3389/fneur.2020.00978 33013647 PMC7506051

[R59] Erny D, Dokalis N, Mezo C, Castoldi A, Mossad O, Staszewski O, Frosch M, Villa M, Fuchs V, Mayer A, Neuber J, Sosat J, Tholen S, Schilling O, Vlachos A, Blank T, Gomez de Aguero M, Macpherson AJ., Pearce EJ and Prinz M (2021) Microbiota-derived acetate enables the metabolic fitness of the brain innate immune system during health and disease. Cell Metabolism, 33(11), 2260–2276 e2267. 10.1016/j.cmet.2021.10.010 34731656

[R60] Erny D, Hrabe de Angelis AL, Jaitin D, Wieghofer P, Staszewski O, David E, Keren-Shaul H, Mahlakoiv T, Jakobshagen K, Buch T, Schwierzeck V, Utermohlen O, Chun E, Garrett WS, McCoy KD, Diefenbach A, Staeheli P, Stecher B, Amit I and Prinz M (2015) Host microbiota constantly control maturation and function of microglia in the CNS. Nature Neuroscience 18(7), 965–977. 10.1038/nn.4030 26030851 PMC5528863

[R61] Espinosa-Oliva AM, Laza RR, Soto MS, Serrano AB, Perez AIR, Ceballos MAR, Revilla JG, Pavon MS, Serres S, Economopoulus V, Vazquez AEC, Carretero MDV, Miranda PG, Klementieva O, Martin MJO, Deierborg T, Infante ER, Sibson NR, Garcia JLL, de la Quintana AM, Rubio MJP, Carmona AJH, Recio JLV and de Pablos RM (2022) Inflammatory bowel disease induces α-synuclein aggregation in gut and brain. 79 bioRxiv, 2022.2001.2026.477259. 10.1101/2022.01.26.477259

[R62] Fransen F, van Beek AA, Borghuis T, Aidy SE, Hugenholtz F, van der Gaast-de Jongh C, Savelkoul HFJ, De Jonge MI, Boekschoten MV, Smidt H, Faas MM and de Vos P (2017) Aged gut microbiota contributes to systemical inflammaging after transfer to germ-free mice. Frontiers in Immunology 8, 1385. 10.3389/fimmu.2017.01385 29163474 PMC5674680

[R63] Fulling C, Dinan TG and Cryan JF (2019) Gut microbe to brain signaling: What happens in vagus. Neuron 101(6), 998–1002. 10.1016/j.neuron.2019.02.008 30897366

[R64] Gardener SL, Rainey-Smith SR, Barnes MB, Sohrabi HR, Weinborn M, Lim YY, Harrington K, Taddei K, Gu Y, Rembach A, Szoeke C, Ellis KA, Masters CL, Macaulay SL, Rowe CC, Ames D, Keogh JB, Scarmeas N and Martins RN (2015) Dietary patterns and cognitive decline in an Australian study of ageing. Molecular Psychiatry 20(7), 860–866. 10.1038/mp.2014.79 25070537

[R65] Ghosh TS, Rampelli S, Jeffery IB, Santoro A, Neto M, Capri M, Giampieri E, Jennings A, Candela M, Turroni S, Zoetendal EG, Hermes GDA, Elodie C, Meunier N, Brugere CM, Pujos-Guillot E, Berendsen AM, De Groot LCPGM, Feskins EJM, Kaluza J, Pietruszka B, Bielak MJ, Comte B, Maijo-Ferre M, Nicoletti C, De Vos WM, Fairweather-Tait S, Cassidy A, Brigidi P, Franceschi C and O’Toole PW (2020) Mediterranean diet intervention alters the gut microbiome in older people reducing frailty and improving health status: The NU-AGE 1-year dietary intervention across five European countries. Gut 69(7), 1218–1228. 10.1136/gutjnl-2019-319654 32066625 PMC7306987

[R66] Ghosh TS, Shanahan F and O’Toole PW (2022a) Toward an improved definition of a healthy microbiome for healthy aging. Nature Aging 2, 1054–1069. 10.1038/s43587-022-00306-9 37118093 PMC10154212

[R67] Ghosh TS, Shanahan F and O’Toole PW (2022b) The gut microbiome as a modulator of healthy ageing. Nature Reviews Gastroenterology & Hepatology 19, 565–584. 10.1038/s41575-022-00605-x 35468952 PMC9035980

[R68] Gibson GR, Hutkins R, Sanders ME, Prescott SL, Reimer RA, Salminen SJ, Scott K, Stanton C, Swanson KS, Cani PD, Verbeke K and Reid G (2017) Expert consensus document: The International Scientific Association for Probiotics and Prebiotics (ISAPP) consensus statement on the definition and scope of prebiotics. Nature Reviews Gastroenterology & Hepatology 14(8), 491–502. 10.1038/nrgastro.2017.75 28611480

[R69] Govindarajan N, Agis-Balboa RC, Walter J, Sananbenesi F and Fischer A (2011) Sodium butyrate improves memory function in an Alzheimer’s disease mouse model when administered at an advanced stage of disease progression. Journal of Alzheimer’s Disease 26(1), 187–197. 10.3233/JAD-2011-110080 21593570

[R70] Grabrucker S, Marizzoni M, Silajdžić E, Lopizzo N, Mombelli E, Nicolas S, Dohm-Hansen S, Scassellati C, Moretti DV, Rosa M, Hoffmann K, English JA, Lavelle A, O’Neill C, Thuret S, Cattaneo A and Nolan YM (2022) Faecal microbiota transplantation from Alzheimer’s participants induces impairments in neurogenesis and cognitive behaviours in rats. bioRxiv, 119 2022.2011.2004.515189. 10.1101/2022.11.04.515189

[R71] Guilherme MDS, Nguyen VTT, Reinhardt C and Endres K (2021) Impact of gut microbiome manipulation in 5xFAD mice on Alzheimer’s disease-like pathology. Microorganisms 9(4), 815. 10.3390/microorganisms9040815 33924322 PMC8069338

[R72] Guo MPJ, Huang X, Xiao L, Huang F and Zuo Z (2021) Gut microbiome features of Chinese patients newly diagnosed with Alzheimer’s disease or mild cognitive impairment. Journal of Alzheimer’s Disease 80(1), 299–310. 10.3233/JAD-201040 33523001

[R73] Guzzetta KE, Cryan JF and O’Leary OF (2022) Microbiota-gut-brain axis regulation of adult hippocampal neurogenesis. Brain Plasticity 8, 97–119. 10.3233/BPL-220141 36448039 PMC9661352

[R74] Hang S, Paik D, Yao L, Kim E, Trinath J, Lu J, Ha S, Nelson BN, Kelly SP, Wu L, Zheng Y, Longman RS, Rastinejad F, Devlin AS, Krout MR, Fischbach MA, Littman DR and Huh JR (2019) Bile acid metabolites control TH17 and Treg cell differentiation. Nature 576(7785), 143–148. 10.1038/s41586-019-1785-z 31776512 PMC6949019

[R75] Harach T, Marungruang N, Duthilleul N, Cheatham V, Mc Coy KD, Frisoni G, Neher JJ, Fåk F, Jucker M, Lasser T and Bolmont T (2017) Reduction of Abeta amyloid pathology in APPPS1 transgenic mice in the absence of gut microbiota. Scientific Reports 7(1), 41802. 10.1038/srep41802 28176819 PMC5297247

[R76] Hazan S (2020) Rapid improvement in Alzheimer’s disease symptoms following fecal microbiota transplantation: A case report. The Journal of International Medical Research 48(6), 300060520925930. 10.1177/0300060520925930 32600151 PMC7328362

[R77] Hibberd PL, Kleimola L, Fiorino AM, Botelho C, Haverkamp M, Andreyeva I, Poutsiaka D, Fraser C, Solano-Aguilar G and Snydman DR (2014) No evidence of harms of probiotic *Lactobacillus rhamnosus* GG ATCC 53103 in healthy elderly-a phase I open label study to assess safety, tolerability and cytokine responses. PLoS One 9(12), e113456. 10.1371/journal.pone.0113456 25438151 PMC4249962

[R78] Hill C (2020) You have the microbiome you deserve. Gut Microbiome 1, e3. 10.1017/gmb.2020.3 PMC11406403 39296724

[R79] Hill C, Guarner F, Reid G, Gibson GR, Merenstein DJ, Pot B, Morelli L, Canani RB, Flint HJ, Salminen S, Calder PC and Sanders ME (2014) Expert consensus document. The International Scientific Association for Probiotics and Prebiotics consensus statement on the scope and appropriate use of the term probiotic. Nature Reviews Gastroenterology & Hepatology 11(8), 506–514. 10.1038/nrgastro.2014.66 24912386

[R80] Hwang YH, Park S, Paik JW, Chae SW, Kim DH, Jeong DG, Ha E, Kim M, Hong G, Park SH, Jung SJ, Lee SM, Na KH, Kim J and Chung YC (2019) Efficacy and safety of Lactobacillus plantarum C29-fermented soybean (DW2009) in individuals with mild cognitive impairment: A 12-week, multi-center, randomized, double-blind, placebo-controlled clinical trial. Nutrients 11(2), 305. 10.3390/nu11020305 30717153 PMC6412773

[R81] Jeffery IB, Lynch DB and O’Toole PW (2016) Composition and temporal stability of the gut microbiota in older persons. The ISME Journal 10(1), 170–182. 10.1038/ismej.2015.88 26090993 PMC4681863

[R82] Kaur H, Nookala S, Singh S, Mukundan S, Nagamoto-Combs K and Combs CK (2021) Sex-dependent effects of intestinal microbiome manipulation in a mouse model of Alzheimer’s disease. Cell 10(9), 2370. 10.3390/cells10092370 PMC8469717 34572019

[R83] Kim BS, Choi CW, Shin H, Jin SP, Bae JS, Han M, Seo EY, Chun J and Chung JH (2019a) Comparison of the gut microbiota of centenarians in longevity villages of South Korea with those of other age groups. Journal of Microbiology and Biotechnology 29(3), 429–440. 10.4014/jmb.1811.11023 30661321

[R84] Kim N, Ho Jeon S, Gyoung Ju I, Sung Gee M, Do J, Sook Oh M and Kil Lee J (2021) Transplantation of gut microbiota derived from Alzheimer’s disease mouse model impairs memory function and neurogenesis in C57BL/6 mice. Brain, Behavior, and Immunity 98, 357–365. 10.1016/j.bbi.2021.09.002 34500036

[R85] Kim S, Kwon SH, Kam TI, Panicker N, Karuppagounder SS, Lee S, Lee JH, Kim WR, Kook M, Foss CA, Shen C, Lee H, Kulkarni S, Pasricha PJ, Lee G, Pomper MG, Dawson VL, Dawson TM and Ko HS (2019b) Transneuronal propagation of pathologic α-synuclein from the gut to the brain models Parkinson’s disease. Neuron, 103(4), 627–641.e7. 10.1016/j.neuron.2019.05.035 31255487 PMC6706297

[R86] Kincaid HJ, Nagpal R and Yadav H (2021) Diet-microbiota-brain axis in Alzheimer’s disease. Annals of Nutrition & Metabolism 77(Suppl 2), 21–27. 10.1159/000515700 33906194 PMC10202336

[R87] Kobayashi Y, Kuhara T, Oki M and Xiao JZ (2019) Effects of *Bifidobacterium breve* A1 on the cognitive function of older adults with memory complaints: A randomised, double-blind, placebo-controlled trial. Beneficial Microbes 10(5), 511–520. 10.3920/BM2018.0170 31090457

[R88] Kobayashi Y, Sugahara H, Shimada K, Mitsuyama E, Kuhara T, Yasuoka A, Kondo T, Abe K and Xiao JZ (2017) Therapeutic potential of *Bifidobacterium breve* strain A1 for preventing cognitive impairment in Alzheimer’s disease. Scientific Reports 7 (1), 13510. 10.1038/s41598-017-13368-2 29044140 PMC5647431

[R89] Koenig JE, Spor A, Scalfone N, Fricker AD, Stombaugh J, Knight R, Angenent LT and Ley RE (2011) Succession of microbial consortia in the developing infant gut microbiome. Proceedings of the National Academy of Sciences 108(Suppl_1), 4578–4585. 10.1073/pnas.1000081107 PMC3063592 20668239

[R90] Kong F, Hua Y, Zeng B, Ning R, Li Y and Zhao J (2016) Gut microbiota signatures of longevity. Current Biology 26(18),R832–R833. 10.1016/j.cub.2016.08.015 27676296

[R91] Kozareva DA, Cryan JF and Nolan YM (2019) Born this way: Hippocampal neurogenesis across the lifespan. Aging Cell 18(5), e13007. 10.1111/acel.13007 31298475 PMC6718573

[R92] Kuai XY, Yao XH, Xu LJ, Zhou YQ, Zhang LP, Liu Y, Pei SF and Zhou CL (2021) Evaluation of fecal microbiota transplantation in Parkinson’s disease patients with constipation. Microbial Cell Factories 20(1), 98. 10.1186/s12934-021-01589-0 33985520 PMC8120701

[R93] Kubinyi E, Bel Rhali S, Sandor S, Szabo A and Felfoldi T (2020) Gut microbiome composition is associated with age and memory performance in pet dogs. Animals (Basel) 10(9), 1488. 10.3390/ani10091488 32846928 PMC7552338

[R94] Kundu P, Lee HU, Garcia-Perez I, Tay EXY, Kim H, Faylon LE, Martin KA, Purbojati R, Drautz-Moses DI, Ghosh S, Nicholson JK, Schuster S, Holmes E and Pettersson S (2019) Neurogenesis and prolongevity signaling in young germ-free mice transplanted with the gut microbiota of old mice. Science Translational Medicine 11(518), eaau4760. 10.1126/scitranslmed.aau4760 31723038

[R95] Labrousse VF, Nadjar A, Joffre C, Costes L, Aubert A, Gregoire S, Bretillon L and Laye S (2012) Short-term long chain omega3 diet protects from neuroinflammatory processes and memory impairment in aged mice. PLoS One 7(5), e36861. 10.1371/journal.pone.0036861 22662127 PMC3360741

[R96] Lassmann H (2018) Pathogenic mechanisms associated with different clinical courses of multiple sclerosis. Frontiers in Immunology 9, 3116. 10.3389/fimmu.2018.03116 30687321 PMC6335289

[R97] Leblhuber F, Steiner K, Schuetz B, Fuchs D and Gostner JM (2018) Probiotic supplementation in patients with Alzheimer’s dementia – An explorative intervention study. Current Alzheimer Research 15(12), 1106–1113. 10.2174/1389200219666180813144834 30101706 PMC6340155

[R98] Lee HY, Lee SH, Lee JH, Lee WJ and Min KJ (2019) The role of commensal microbes in the lifespan of *Drosophila melanogaster*. Aging (Albany NY) 11(13), 4611–4640. 10.18632/aging.102073 31299010 PMC6660043

[R99] Leite G, Pimentel M, Barlow GM, Chang C, Hosseini A, Wang J, Parodi G, Sedighi R, Rezaie A and Mathur R (2021) Age and the aging process significantly alter the small bowel microbiome. Cell Reports 36(13), 109765. 10.1016/j.celrep.2021.109765 34592155

[R100] Leite G, Pimentel M, Barlow GM and Mathur R (2022) The small bowel microbiome changes significantly with age and aspects of the ageing process. Microbial Cell 9(1), 21–23. 10.15698/mic2022.01.768 35083314 PMC8717087

[R101] Lendahl U, Nilsson P and Betsholtz C (2019) Emerging links between cerebrovascular and neurodegenerative diseases – A special role for pericytes. EMBO Reports 20(11), e48070. 10.15252/embr.201948070 31617312 PMC6831996

[R102] Li B, He Y, Ma J, Huang P, Du J, Cao L, Wang Y, Xiao Q, Tang H and Chen S (2019) Mild cognitive impairment has similar alterations as Alzheimer’s disease in gut microbiota. Alzheimers Dementia 15(10), 1357–1366. 10.1016/j.jalz.2019.07.002 31434623

[R103] Li Y, Ning L, Yin Y, Wang R, Zhang Z, Hao L, Wang B, Zhao X, Yang X, Yin L, Wu S, Guo D and Zhang C (2020) Age-related shifts in gut microbiota contribute to cognitive decline in aged rats. Aging (Albany NY) 12(9), 7801–7817. 10.18632/aging.103093 32357144 PMC7244050

[R104] Liu B, Fang F, Pedersen NL, Tillander A, Ludvigsson JF, Ekbom A, Svenningsson P, Chen H, and Wirdefeldt K (2017) Vagotomy and Parkinson disease: A Swedish register-based matched-cohort study. Neurology 88(21), 1996–2002. 10.1212/WNL.0000000000003961 28446653 PMC5440238

[R105] Liu KH, Owens JA, Saeedi B, Cohen CE, Bellissimo MP, Naudin C, Darby T, Druzak S, Maner-Smith K, Orr M, Hu X, Fernandes J, Camacho MC, Hunter-Chang S, VanInsberghe D, Ma C, Ganesh T, Yeligar SM, Uppal K, Go YM, Alvarez JA, Vos MB, Ziegler TR, Woodworth MH, Kraft CS, Jones RM, Ortlund E, Neish AS and Jones DP (2021) Microbial metabolite delta-valerobetaine is a diet-dependent obesogen. Nature Metabolism 3(12), 1694–1705. 10.1038/s42255-021-00502-8 PMC8711632 34931082

[R106] Liu S, Rezende RM, Moreira TG, Tankou SK, Cox LM, Wu M, Song A, Dhang FH, Wei Z, Costamagna G and Weiner HL (2019) Oral administration of miR-30d from feces of MS patients suppresses MS-like symptoms in mice by expanding *Akkermansia muciniphila*. Cell Host & Microbe 26(6), 779–794.e8. 10.1016/j.chom.2019.10.008 31784260 PMC6948921

[R107] Louzada ER and Ribeiro SML (2020) Synbiotic supplementation, systemic inflammation, and symptoms of brain disorders in elders: A secondary study from a randomized clinical trial. Nutritional Neuroscience 23(2), 93–100. 10.1080/1028415X.2018.1477349 29788823

[R108] Lu CS, Chang HC, Weng YH, Chen CC, Kuo YS and Tsai YC (2021) The add-on effect of *Lactobacillus plantarum* PS128 in patients with Parkinson’s disease: A pilot study. Frontiers in Nutrition 8, 650053. 10.3389/fnut.2021.650053 34277679 PMC8277995

[R109] MahmoudianDehkordi S, Arnold M, Nho K, Ahmad S, Jia W, Xie G, Louie G, Kueider-Paisley A, Moseley MA, Thompson JW, St John Williams L, Tenenbaum JD, Blach C, Baillie R, Han X, Bhattacharyya S, Toledo JB, Schafferer S, Klein S, Koal T, Risacher SL, Kling MA, Motsinger-Reif A, Rotroff DM, Jack J, Hankemeier T, Bennett DA, De Jager PL, Trojanowski JQ, Shaw LM, Weiner MW, Doraiswamy PM, van Duijn CM, Saykin AJ, Kastenmuller G, Kaddurah-Daouk R, Alzheimer’s Disease Neuroimaging Initiative and the Alzheimer’s Disease Metabolomics Consortium (2019) Altered bile acid profile associates with cognitive impairment in Alzheimer’s disease – An emerging role for gut microbiome. Alzheimers & Dementia 15(1), 76–92. 10.1016/j.jalz.2018.07.217 PMC6487485 30337151

[R110] Maldonado-Gomez MX, Martinez I, Bottacini F, O’Callaghan A, Ventura M, van Sinderen D, Hillmann B, Vangay P, Knights D, Hutkins RW and Walter J (2016) Stable engraftment of *Bifidobacterium longum* AH1206 in the human gut depends on individualized features of the resident microbiome. Cell Host & Microbe 20(4), 515–526. 10.1016/j.chom.2016.09.001 27693307

[R111] Marquez EJ, Chung CH, Marches R, Rossi RJ, Nehar-Belaid D, Eroglu A, Mellert DJ, Kuchel GA, Banchereau J and Ucar D (2020) Sexual-dimorphism in human immune system aging. Nature Communications 11(1), 751. 10.1038/s41467-020-14396-9 PMC7005316 32029736

[R112] Marseglia A, Xu W, Fratiglioni L, Fabbri C, Berendsen AAM, Bialecka-Debek A, Jennings A, Gillings R, Meunier N, Caumon E, Fairweather-Tait S, Pietruszka B, De Groot L, Santoro A and Franceschi C (2018) Effect of the NU-AGE diet on cognitive functioning in older adults: A randomized controlled trial. Frontiers in Physiology 9, 349. 10.3389/fphys.2018.00349 29670545 PMC5893841

[R113] Matheoud D, Cannon T, Voisin A, Penttinen AM, Ramet L, Fahmy AM, Ducrot C, Laplante A, Bourque MJ, Zhu L, Cayrol R, Le Campion A, McBride HM, Gruenheid S, Trudeau LE and Desjardins M (2019) Intestinal infection triggers Parkinson’s disease-like symptoms in Pink1(−/−) mice. Nature 571(7766), 565–569. 10.1038/s41586-019-1405-y 31316206

[R114] Matt SM, Allen JM, Lawson MA, Mailing LJ, Woods JA and Johnson RW (2018) Butyrate and dietary soluble fiber improve neuroinflammation associated with aging in mice. Frontiers in Immunology 9, 1832. 10.3389/fimmu.2018.01832 30154787 PMC6102557

[R115] Mayneris-Perxachs J, Castells-Nobau A, Arnoriaga-Rodríguez M, Garre-Olmo J, Puig J, Ramos R, Martínez-Hernández F, Burokas A, Coll C, Moreno-Navarrete JM, Zapata-Tona C, Pedraza S, Pérez-Brocal V, Ramió-Torrentà L, Ricart W, Moya A, Martínez-García M, Maldonado R and Fernández-Real J-M(2022) Caudovirales bacteriophages are associated with improved executive function and memory in flies, mice, and humans. Cell Host & Microbe 30, 340–356.e8. 10.1016/j.chom.2022.01.013 35176247

[R116] Mehta RS, Lochhead P, Wang Y, Ma W, Nguyen LH, Kochar B, Huttenhower C, Grodstein F and Chan AT (2022) Association of midlife antibiotic use with subsequent cognitive function in women. PLoS One 17(3), e0264649. 10.1371/journal.pone.0264649 35320274 PMC8942267

[R117] Mezö C, Dokalis N, Mossad O, Staszewski O, Neuber J, Yilmaz B, Schnepf D, de Agüero MG, Ganal-Vonarburg SC, Macpherson AJ, Meyer-Luehmann M, Staeheli P, Blank T, Prinz M and Erny D (2020) Different effects of constitutive and induced microbiota modulation on microglia in a mouse model of Alzheimer’s disease. Acta Neuropathologica Communications 8(1), 119. 10.1186/s40478-020-00988-5 32727612 PMC7389451

[R118] Minter MR, Hinterleitner R, Meisel M, Zhang C, Leone V, Zhang X, Oyler-Castrillo P, Zhang X, Musch MW, Shen X, Jabri B, Chang EB, Tanzi RE and Sisodia SS (2017) Antibiotic-induced perturbations in microbial diversity during post-natal development alters amyloid pathology in an aged APP(SWE)/PS1(ΔE9) murine model of Alzheimer’s disease. Scientific Reports 7(1), 10411. 10.1038/s41598-017-11047-w 28874832 PMC5585265

[R119] Minter MR, Zhang C, Leone V, Ringus DL, Zhang X, Oyler-Castrillo P, Musch MW, Liao F, Ward JF, Holtzman DM, Chang EB, Tanzi RE and Sisodia SS (2016) Antibiotic-induced perturbations in gut microbial diversity influences neuroinflammation and amyloidosis in a murine model of Alzheimer’s disease. Scientific Reports 6, 30028. 10.1038/srep30028 27443609 PMC4956742

[R120] Mirza A, Forbes JD, Zhu F, Bernstein CN, Van Domselaar G, Graham M, Waubant E and Tremlett H (2020) The multiple sclerosis gut microbiota: A systematic review. Multiple Sclerosis and Related Disorders 37, 101427. 10.1016/j.msard.2019.101427 32172998

[R121] Mondal B, Choudhury S, Banerjee R, Roy A, Chatterjee K, Basu P, Singh R, Halder S, Shubham S, Baker SN, Baker MR and Kumar H (2021) Non-invasive vagus nerve stimulation improves clinical and molecular biomarkers of Parkinson’s disease in patients with freezing of gait. NPJ Parkinsons Disease 7(1), 46. 10.1038/s41531-021-00190-x PMC8160211 34045464

[R122] Montgomery TL, Eckstrom K, Lile KH, Caldwell S, Heney ER, Lahue KG, D’Alessandro A, Wargo MJ and Krementsov DN (2022). Lactobacillus reuteri tryptophan metabolism promotes host susceptibility to CNS autoimmunity. Microbiome 10, 198. 36419205 10.1186/s40168-022-01408-7 PMC9685921

[R123] Moore AR and O’Keeffe ST (1999) Drug-induced cognitive impairment in the elderly. Drugs & Aging 15(1), 15–28. 10.2165/00002512-199915010-00002 10459729

[R124] Mosaferi B, Jand Y and Salari AA (2021) Gut microbiota depletion from early adolescence alters anxiety and depression-related behaviours in male mice with Alzheimer-like disease. Scientific Reports 11(1), 22941. 10.1038/s41598-021-02231-0 34824332 PMC8617202

[R125] Mossad O, Batut B, Yilmaz B, Dokalis N, Mezo C, Nent E, Nabavi LS, Mayer M, Maron FJM, Buescher JM, de Aguero MG, Szalay A, Lammermann T, Macpherson AJ, Ganal-Vonarburg SC, Backofen R, Erny D, Prinz M and Blank T (2022) Gut microbiota drives age-related oxidative stress and mitochondrial damage in microglia via the metabolite N(6)-carboxymethyllysine. Nature Neuroscience 25(3), 295–305. 10.1038/s41593-022-01027-3 35241804

[R126] Mossad O and Blank T (2021) Getting on in old age: How the gut microbiota interferes with brain innate immunity. Frontiers in Cellular Neuroscience 15, 698126. 10.3389/fncel.2021.698126 34295223 PMC8290125

[R127] Mossad O, Nent E, Woltemate S, Folschweiller S, Buescher JM, Schnepf D, Erny D, Staeheli P, Bartos M, Szalay A, Stecher B, Vital M, Sauer JF, Lämmermann T, Prinz M and Blank T (2021) Microbiota-dependent increase in δ-valerobetaine alters neuronal function and is responsible for age-related cognitive decline. Nature Aging 1, 1127–1136. 10.1038/s43587-021-00141-4 37117525

[R128] Murphy DG, DeCarli C, McIntosh AR, Daly E, Mentis MJ, Pietrini P, Szczepanik J, Schapiro MB, Grady CL, Horwitz B, Rapoport SI (1996) Sex differences in human brain morphometry and metabolism: An in vivo quantitative magnetic resonance imaging and positron emission tomography study on the effect of aging. Archives of General Psychiatry 53(7), 585–594. 10.1001/archpsyc.1996.01830070031007 8660125

[R129] Nagpal R, Neth BJ, Wang S, Craft S and Yadav H (2019) Modified mediterranean-ketogenic diet modulates gut microbiome and short-chain fatty acids in association with Alzheimer’s disease markers in subjects with mild cognitive impairment. eBioMedicine 47, 529–542. 10.1016/j.ebiom.2019.08.032 31477562 PMC6796564

[R130] Nau R, Sörgel F and Eiffert H (2010) Penetration of drugs through the blood-cerebrospinal fluid/blood-brain barrier for treatment of central nervous system infections. Clinical Microbiology Reviews 23(4), 858–883. 10.1128/CMR.00007-10 20930076 PMC2952976

[R131] Neuffer J, González-Domínguez R, Lefèvre-Arbogast S, Low DY, Driollet B, Helmer C, Du Preez A, de Lucia C, Ruigrok SR, Altendorfer B, Aigner L, Lucassen PJ, Korosi A, Thuret S, Manach C, Pallàs M, Urpi-Sardà M, Sánchez-Pla A, Andres-Lacueva C and Samieri C (2022) Exploration of the gut–brain axis through metabolomics identifies serum propionic acid associated with higher cognitive decline in older persons. Nutrients, 14(21), 4688. Available at https://www.mdpi.com/2072-6643/14/21/4688 Accessed on the 19th November. 36364950 10.3390/nu14214688 PMC9655149

[R132] Nichols E, Steinmetz JD, Vollset SE, Fukutaki K, Chalek J, Abd-Allah F, Abdoli A, Abualhasan A, Abu-Gharbieh E, Akram TT, Al Hamad H, Alahdab F, Alanezi FM, Alipour V, Almustanyir S, Amu H, Ansari I, Arabloo J, Ashraf T, Astell-Burt T, Ayano G, Ayuso-Mateos JL, Baig AA, Barnett A, Barrow A, Baune BT, Béjot Y, Bezabhe WMM, Bezabih YM, Bhagavathula AS, Bhaskar S, Bhattacharyya K, Bijani A, Biswas A, Bolla SR, Boloor A, Brayne C, Brenner H, Burkart K, Burns RA, Cámera LA, Cao C, Carvalho F, Castro-de-Araujo LFS, Catalá-López F, Cerin E, Chavan PP, Cherbuin N, Chu D-T, Costa VM, Couto RAS, Dadras O, Dai X, Dandona L, Dandona R, De la Cruz-Góngora V, Dhamnetiya D, Dias da Silva D, Diaz D, Douiri A, Edvardsson D, Ekholuenetale M, El Sayed I, El-Jaafary SI, Eskandari K, Eskandarieh S, Esmaeilnejad S, Fares J, Faro A, Farooque U, Feigin VL, Feng X, Fereshtehnejad S-M, Fernandes E, Ferrara P, Filip I, Fillit H, Fischer F, Gaidhane S, Galluzzo L, Ghashghaee A, Ghith N, Gialluisi A, Gilani SA, Glavan I-R, Gnedovskaya EV, Golechha M, Gupta R, Gupta VB, Gupta VK, Haider MR, Hall BJ, Hamidi S, Hanif A, Hankey GJ, Haque S, Hartono RK, Hasaballah AI, Hasan MT, Hassan A, Hay SI, Hayat K, Hegazy MI, Heidari G, Heidari-Soureshjani R, Herteliu C, Househ M, Hussain R, Hwang B-F, Iacoviello L, Iavicoli I, Ilesanmi OS, Ilic IM, Ilic MD, Irvani SSN, Iso H, Iwagami M, Jabbarinejad R, Jacob L, Jain V, Jayapal SK, Jayawardena R, Jha RP, Jonas JB, Joseph N, Kalani R, Kandel A, Kandel H, Karch A, Kasa AS, Kassie GM, Keshavarz P, Khan MAB, Khatib MN, Khoja TAM, Khubchandani J, Kim MS, Kim YJ, Kisa A, Kisa S, Kivimäki M, Koroshetz WJ, Koyanagi A, Kumar GA, Kumar M, Lak HM, Leonardi M, Li B, Lim SS, Liu X, Liu Y, Logroscino G, Lorkowski S, Lucchetti G, Lutzky Saute R, Magnani FG, Malik AA, Massano J, Mehndiratta MM, Menezes RG, Meretoja A, Mohajer B, Mohamed Ibrahim N, Mohammad Y, Mohammed A, Mokdad AH, Mondello S, Moni MAA, Moniruzzaman M, Mossie TB, Nagel G, Naveed M, Nayak VC, Neupane Kandel S, Nguyen TH, Oancea B, Otstavnov N, Otstavnov SS, Owolabi MO, Panda-Jonas S, Pashazadeh Kan F, Pasovic M, Patel UK, Pathak M, Peres MFP, Perianayagam A, Peterson CB, Phillips MR, Pinheiro M, Piradov MA, Pond CD, Potashman MH, Pottoo FH, Prada SI, Radfar A, Raggi A, Rahim F, Rahman M, Ram P, Ranasinghe P, Rawaf DL, Rawaf S, Rezaei N, Rezapour A, Robinson SR, Romoli M, Roshandel G, Sahathevan R, Sahebkar A, Sahraian MA, Sathian B, Sattin D, Sawhney M, Saylan M, Schiavolin S, Seylani A, Sha F, Shaikh MA, Shaji KS, Shannawaz M, Shetty JK, Shigematsu M, Shin JI, Shiri R, Silva DAS, Silva JP, Silva R, Singh JA, Skryabin VY, Skryabina AA, Smith AE, Soshnikov S, Spurlock EE, Stein DJ, Sun J, Tabarés-Seisdedos R, Thakur B, Timalsina B, Tovani-Palone MR, Tran BX, Tsegaye GW, Valadan Tahbaz S, Valdez PR, Venketasubramanian N, Vlassov V, Vu GT, Vu LG, Wang Y-P, Wimo A, Winkler AS, Yadav L, Yahyazadeh Jabbari SH, Yamagishi K, Yang L, Yano Y, Yonemoto N, Yu C, Yunusa I, Zadey S, Zastrozhin MS, Zastrozhina A, Zhang Z-J, Murray CJL, Vos T (2022) Estimation of the global prevalence of dementia in 2019 and forecasted prevalence in 2050: An analysis for the global burden of disease study 2019. The Lancet Public Health 7(2), e105–e125. 10.1016/S2468-2667(21)00249-8 34998485 PMC8810394

[R133] Odamaki T, Kato K, Sugahara H, Hashikura N, Takahashi S, Xiao JZ, Abe F and Osawa R (2016) Age-related changes in gut microbiota composition from newborn to centenarian: A cross-sectional study. BMC Microbiology 16, 90. 10.1186/s12866-016-0708-5 27220822 PMC4879732

[R134] Ogbonnaya ES, Clarke G, Shanahan F, Dinan TG, Cryan JF and O’Leary OF (2015) Adult hippocampal neurogenesis is regulated by the microbiome. Biological Psychiatry 78(4), e7–e9. 10.1016/j.biopsych.2014.12.023 25700599

[R135] Olson CA, Vuong HE, Yano JM, Liang QY, Nusbaum DJ and Hsiao EY (2018) The gut microbiota mediates the anti-seizure effects of the ketogenic diet. Cell 174(2), 497. 10.1016/j.cell.2018.06.051 30007420 PMC6062008

[R136] Ooi TC, Meramat A, Rajab NF, Shahar S, Ismail IS, Azam AA and Sharif R (2020) Intermittent fasting enhanced the cognitive function in older adults with mild cognitive impairment by inducing biochemical and metabolic changes: A 3-year progressive study. Nutrients 12(9), 2644. 10.3390/nu12092644 32872655 PMC7551340

[R137] Ou Z, Deng L, Lu Z, Wu F, Liu W, Huang D and Peng Y (2020) Protective effects of *Akkermansia muciniphila* on cognitive deficits and amyloid pathology in a mouse model of Alzheimer’s disease. Nutrition & Diabetes 10(1), 12. 10.1038/s41387-020-0115-8 32321934 PMC7176648

[R138] Pallikkuth S, Mendez R, Russell K, Sirupangi T, Kvistad D, Pahwa R, Villinger F, Banerjee S and Pahwa S (2021) Age associated microbiome and microbial metabolites modulation and its association with systemic inflammation in a rhesus macaque model. Frontiers in Immunology 12:748397 10.3389/fimmu.2021.748397 34737748 PMC8560971

[R139] Pan R-Y, Zhang J, Wang J, Wang Y, Li Z, Liao Y, Liao Y, Zhang C, Liu Z, Song L, Yu J and Yuan Z (2022) Intermittent fasting protects against Alzheimer’s disease in mice by altering metabolism through remodeling of the gut microbiota. Nature Aging 2, 1–16. 10.1038/s43587-022-00311-y 37118092

[R140] Park SH, Lee JH, Shin J, Kim JS, Cha B, Lee S, Kwon KS, Shin YW and Choi SH (2021) Cognitive function improvement after fecal microbiota transplantation in Alzheimer’s dementia patient: A case report. Current Medical Research and Opinion 37(10), 1739–1744. 10.1080/03007995.2021.1957807 34289768

[R141] Parker A, Romano S, Ansorge R, Aboelnour A, Le Gall G, Savva GM, Pontifex MG, Telatin A, Baker D, Jones E, Vauzour D, Rudder S, Blackshaw LA, Jeffery G and Carding SR (2022) Fecal microbiota transfer between young and aged mice reverses hallmarks of the aging gut, eye, and brain. Microbiome 10(1), 68. 10.1186/s40168-022-01243-w 35501923 PMC9063061

[R142] Perez-Pardo P, Kliest T, Dodiya HB, Broersen LM, Garssen J, Keshavarzian A and Kraneveld AD (2017) The gut-brain axis in Parkinson’s disease: Possibilities for food-based therapies. European Journal of Pharmacology 817, 86–95. 10.1016/j.ejphar.2017.05.042 28549787

[R143] Peters R (2006) Ageing and the brain. Postgraduate Medical Journal 82(964), 84–88. 10.1136/pgmj.2005.036665 16461469 PMC2596698

[R144] Petersen RC, Roberts RO, Knopman DS, Geda YE, Cha RH, Pankratz VS, Boeve BF, Tangalos EG, Ivnik RJ and Rocca WA (2010) Prevalence of mild cognitive impairment is higher in men. The Mayo Clinic Study of Aging. Neurology 75(10), 889–897. 10.1212/WNL.0b013e3181f11d85 20820000 PMC2938972

[R145] Provensi G, Schmidt SD, Boehme M, Bastiaanssen TFS, Rani B, Costa A, Busca K, Fouhy F, Strain C, Stanton C, Blandina P, Izquierdo I, Cryan JF, Passani MB (2019) Preventing adolescent stress-induced cognitive and microbiome changes by diet. Proceedings of the National Academy of Sciences of the United States of America 116(19), 9644–9651. 10.1073/pnas.1820832116 31010921 PMC6511019

[R146] Pu A, Lee DSW, Isho B, Naouar I and Gommerman JL (2021) The impact of IgA and the microbiota on CNS Disease. Frontiers in Immunology 12, 742173. 10.3389/fimmu.2021.742173 34603329 PMC8479159

[R147] Ratiner K, Abdeen SK, Goldenberg K and Elinav E (2022) Utilization of host and microbiome features in determination of biological aging. Microorganisms 10(3), 668. 10.3390/microorganisms10030668 35336242 PMC8950177

[R148] Regen T, Isaac S, Amorim A, Nunez NG, Hauptmann J, Shanmugavadivu A, Klein M, Sankowski R, Mufazalov IA, Yogev N, Huppert J, Wanke F, Witting M, Grill A, Galvez EJC, Nikolaev A, Blanfeld M, Prinz I, Schmitt-Kopplin P, Strowig T, Reinhardt C, Prinz M, Bopp T, Becher B, Ubeda C and Waisman A (2021) IL-17 controls central nervous system autoimmunity through the intestinal microbiome. Science Immunology 6(56), eaaz6563. 10.1126/sciimmunol.aaz6563 33547052

[R149] Rei D, Saha S, Haddad M, Haider Rubio A, Perlaza BL, Berard M, Ungeheuer MN, Sokol H and Lledo PM (2022) Age-associated gut microbiota impairs hippocampus-dependent memory in a vagus-dependent manner. JCI Insight 7, e147700. 10.1172/jci.insight.147700 35737457 PMC9462480

[R150] Rio DD, Zimetti F, Caffarra P, Tassotti M, Bernini F, Brighenti F, Zini A and Zanotti I (2017) The gut microbial metabolite trimethylamine-N-oxide is present in human cerebrospinal fluid. Nutrients 9(10), 1053. 10.3390/nu9101053 28937600 PMC5691670

[R151] Romano S, Savva GM, Bedarf JR, Charles IG, Hildebrand F and Narbad A (2021) Meta-analysis of the Parkinson’s disease gut microbiome suggests alterations linked to intestinal inflammation. NPJ Parkinsons Disease 7(1), 27. 10.1038/s41531-021-00156-z PMC7946946 33692356

[R152] Romo-Araiza A, Gutierrez-Salmean G, Galvan EJ, Hernandez-Frausto M, Herrera-Lopez G, Romo-Parra H, Garcia-Contreras V, Fernandez-Presas AM, Jasso-Chavez R, Borlongan CV and Ibarra A (2018) Probiotics and prebiotics as a therapeutic strategy to improve memory in a model of middle-aged rats. Frontiers in Aging Neuroscience 10, 416. 10.3389/fnagi.2018.00416 30618722 PMC6305305

[R153] Rothhammer V, Borucki DM, Tjon EC, Takenaka MC, Chao CC, Ardura-Fabregat A, de Lima KA, Gutierrez-Vazquez C, Hewson P, Staszewski O, Blain M, Healy L, Neziraj T, Borio M, Wheeler M, Dragin LL, Laplaud DA, Antel J, Alvarez JI, Prinz M and Quintana FJ (2018) Microglial control of astrocytes in response to microbial metabolites. Nature 557(7707), 724–728. 10.1038/s41586-018-0119-x 29769726 PMC6422159

[R154] Saffrey MJ (2014) Aging of the mammalian gastrointestinal tract: A complex organ system. Age (Dordrecht, Netherlands) 36(3),9603. 10.1007/s11357-013-9603-2 24352567 PMC4082571

[R155] Saji N, Niida S, Murotani K, Hisada T, Tsuduki T, Sugimoto T, Kimura A, Toba K and Sakurai T (2019) Analysis of the relationship between the gut microbiome and dementia: A cross-sectional study conducted in Japan. Scientific Reports 9(1), 1008. 10.1038/s41598-018-38218-7 30700769 PMC6353871

[R156] Salazar N, Valdes-Varela L, Gonzalez S, Gueimonde M and de Los Reyes-Gavilan CG (2017) Nutrition and the gut microbiome in the elderly. Gut Microbes 8(2), 82–97. 10.1080/19490976.2016.1256525 27808595 PMC5390822

[R157] Sampson TR, Debelius JW, Thron T, Janssen S, Shastri GG, Ilhan ZE, Challis C, Schretter CE, Rocha S, Gradinaru V, Chesselet MF, Keshavarzian A, Shannon KM, Krajmalnik-Brown R, Wittung-Stafshede P, Knight R and Mazmanian SK (2016) Gut microbiota regulate motor deficits and neuroinflammation in a model of Parkinson’s disease. Cell 167(6), 1469–1480.e12. 10.1016/j.cell.2016.11.018 27912057 PMC5718049

[R158] Sanborn V, Azcarate-Peril MA, Updegraff J, Manderino L and Gunstad J (2020) Randomized clinical trial examining the impact of *Lactobacillus rhamnosus* GG probiotic supplementation on cognitive functioning in middle-aged and older adults. Neuropsychiatric Disease and Treatment 16, 2765–2777. 10.2147/NDT.S270035 33223831 PMC7671471

[R159] Sato Y, Atarashi K, Plichta DR, Arai Y, Sasajima S, Kearney SM, Suda W, Takeshita K, Sasaki T, Okamoto S, Skelly AN, Okamura Y, Vlamakis H, Li Y, Tanoue T, Takei H, Nittono H, Narushima S, Irie J, Itoh H, Moriya K, Sugiura Y, Suematsu M, Moritoki N, Shibata S, Littman DR, Fischbach MA, Uwamino Y, Inoue T, Honda A, Hattori M, Murai T, Xavier RJ, Hirose N and Honda K (2021) Novel bile acid biosynthetic pathways are enriched in the microbiome of centenarians. Nature. 10.1038/s41586-021-03832-5 34325466

[R160] Schliamser SE, Cars O and Norrby SR (1991) Neurotoxicity of beta-lactam antibiotics: Predisposing factors and pathogenesis. The Journal of Antimicrobial Chemotherapy 27(4), 405–425. 10.1093/jac/27.4.405 1856121

[R161] Schultz M, Linde HJ, Lehn N, Zimmermann K, Grossmann J, Falk W and Scholmerich J (2003) Immunomodulatory consequences of oral administration of *Lactobacillus rhamnosus* strain GG in healthy volunteers. The Journal of Dairy Research 70(2), 165–173. 10.1017/s0022029903006034 12800870

[R162] Scott KA, Ida M, Peterson VL, Prenderville JA, Moloney GM, Izumo T, Murphy K, Murphy A, Ross RP, Stanton C, Dinan TG, Cryan JF (2017) Revisiting Metchnikoff: Age-related alterations in microbiota-gut-brain axis in the mouse. Brain, Behavior, and Immunity 65, 20–32. 10.1016/j.bbi.2017.02.004 28179108

[R163] Shanahan F, Ghosh TS and O’Toole PW (2021) The healthy microbiome-what is the definition of a healthy gut microbiome? Gastroenterology 160(2), 483–494. 10.1053/j.gastro.2020.09.057 33253682

[R164] Shepherd ES, DeLoache WC, Pruss KM, Whitaker WR and Sonnenburg JL (2018) An exclusive metabolic niche enables strain engraftment in the gut microbiota. Nature 557(7705), 434–438. 10.1038/s41586-018-0092-4 29743671 PMC6126907

[R165] Shoemark DK and Allen SJ (2015) The microbiome and disease: Reviewing the links between the oral microbiome, aging, and Alzheimer’s disease. Journal of Alzheimer’s Disease 43(3), 725–738. 10.3233/JAD-141170 25125469

[R166] Smith P, Willemsen D, Popkes M, Metge F, Gandiwa E, Reichard M and Valenzano DR (2017) Regulation of life span by the gut microbiota in the short-lived African turquoise killifish. Elife 6. 10.7554/eLife.27014 PMC5566455 28826469

[R167] Soenen S, Rayner CK, Jones KL and Horowitz M (2016) The ageing gastrointestinal tract. Current Opinion in Clinical Nutrition and Metabolic Care 19(1), 12–18. 10.1097/mco.0000000000000238 26560524

[R168] Sorbara MT and Pamer EG (2022) Microbiome-based therapeutics. Nature Reviews Microbiology, 20(6):365–380. 10.1038/s41579-021-00667-9 34992261

[R169] Spychala MS, Venna VR, Jandzinski M, Doran SJ, Durgan DJ, Ganesh BP, Ajami NJ, Putluri N, Graf J, Bryan RM and McCullough LD (2018) Age-related changes in the gut microbiota influence systemic inflammation and stroke outcome. Annals of Neurology 84(1), 23–36. 10.1002/ana.25250 29733457 PMC6119509

[R170] Stebegg M, Silva-Cayetano A, Innocentin S, Jenkins TP, Cantacessi C, Gilbert C and Linterman MA (2019) Heterochronic faecal transplantation boosts gut germinal centres in aged mice. Nature Communications 10(1), 2443. 10.1038/s41467-019-10430-7 PMC6547660 31164642

[R171] Stilling RM, van de Wouw M, Clarke G, Stanton C, Dinan TG and Cryan JF (2016) The neuropharmacology of butyrate: The bread and butter of the microbiota-gut-brain axis? Neurochemistry International 99, 110–132. 10.1016/j.neuint.2016.06.011 27346602

[R172] Suhocki PV, Ronald JS, Diehl AME, Murdoch DM and Doraiswamy PM (2022) Probing gut-brain links in Alzheimer’s disease with rifaximin. Alzheimer’s & Dementia (New York, N. Y.) 8(1), e12225. 10.1002/trc2.12225 PMC8804600 35128026

[R173] Sun J, Xu J, Ling Y, Wang F, Gong T, Yang C, Ye S, Ye K, Wei D, Song Z, Chen D and Liu J (2019) Fecal microbiota transplantation alleviated Alzheimer’s disease-like pathogenesis in APP/PS1 transgenic mice. Translational Psychiatry 9(1), 189. 10.1038/s41398-019-0525-3 31383855 PMC6683152

[R174] Sung HY, Park JW and Kim JS (2014) The frequency and severity of gastrointestinal symptoms in patients with early Parkinson’s disease. Journal of Movement Disorders 7(1), 7–12. 10.14802/jmd.14002 24926404 PMC4051727

[R175] Svensson E, Horvath-Puho E, Thomsen RW, Djurhuus JC, Pedersen L, Borghammer P and Sorensen HT (2015) Vagotomy and subsequent risk of Parkinson’s disease. Annals of Neurology 78(4), 522–529. 10.1002/ana.24448 26031848

[R176] Tamtaji OR, Heidari-Soureshjani R, Mirhosseini N, Kouchaki E, Bahmani F, Aghadavod E, Tajabadi-Ebrahimi M and Asemi Z (2019) Probiotic and selenium co-supplementation, and the effects on clinical, metabolic and genetic status in Alzheimer’s disease: A randomized, double-blind, controlled trial. Clinical Nutrition 38(6), 2569–2575. 10.1016/j.clnu.2018.11.034 30642737

[R177] Tansey MG, Wallings RL, Houser MC, Herrick MK, Keating CE and Joers V (2022) Inflammation and immune dysfunction in Parkinson disease. Nature Reviews Immunology 22, 657–673. 10.1038/s41577-022-00684-6 PMC8895080 35246670

[R178] Tazume S, Umehara K, Matsuzawa H, Aikawa H, Hashimoto K and Sasaki S (1991) Effects of germfree status and food restriction on longevity and growth of mice. Jikken Dobutsu 40(4), 517–522. 10.1538/expanim1978.40.4_517 1748169

[R179] Teng Y, Mu J, Xu F, Zhang X, Sriwastva MK, Liu QM, Li X, Lei C, Sundaram K, Hu X, Zhang L, Park JW, Hwang JY, Rouchka EC, Zhang X, Yan J, Merchant ML and Zhang HG (2022) Gut bacterial isoamylamine promotes age-related cognitive dysfunction by promoting microglial cell death. Cell Host & Microbe 30, 944–960.e8. 10.1016/j.chom.2022.05.005 35654045 PMC9283381

[R180] TheouO, JayanamaK, Fernandez-GarridoJ, BuiguesC, PruimboomL, HooglandAJ, Navarro-MartinezR, RockwoodK and CauliO (2019) Can a prebiotic formulation reduce frailty levels in older people? The Journal of Frailty & Aging 8(1), 48–52. 10.14283/jfa.2018.3930734832

[R181] Thevaranjan N, Puchta A, Schulz C, Naidoo A, Szamosi JC, Verschoor CP, Loukov D, Schenck LP, Jury J, Foley KP, Schertzer JD, Larche MJ, Davidson DJ, Verdu EF, Surette MG and Bowdish DME (2017) Age-associated microbial dysbiosis promotes intestinal permeability, systemic inflammation, and macrophage dysfunction. Cell Host & Microbe 21(4), 455–466.e4. 10.1016/j.chom.2017.03.002 28407483 PMC5392495

[R182] Touyarot K, Bonhomme D, Roux P, Alfos S, Lafenetre P, Richard E, Higueret P and Pallet V (2013) A mid-life vitamin a supplementation prevents age-related spatial memory deficits and hippocampal neurogenesis alterations through CRABP-I. PLoS One 8(8), e72101. 10.1371/journal.pone.0072101 23977218 PMC3747058

[R183] Trollor JN, Smith E, Baune BT, Kochan NA, Campbell L, Samaras K, Crawford J, Brodaty H and Sachdev P (2010) Systemic inflammation is associated with MCI and its subtypes: The Sydney memory and aging study. Dementia and Geriatric Cognitive Disorders 30(6), 569–578. 10.1159/000322092 21252552

[R184] Tuikhar N, Keisam S, Labala RK, Imrat RP, Arunkumar MC, Ahmed G, Biagi E, Jeyaram K (2019) Comparative analysis of the gut microbiota in centenarians and young adults shows a common signature across genotypically non-related populations. Mechanisms of Ageing and Development 179, 23–35. 10.1016/j.mad.2019.02.001 30738080

[R185] Ueda A, Shinkai S, Shiroma H, Taniguchi Y, Tsuchida S, Kariya T, Kawahara T, Kobayashi Y, Kohda N, Ushida K, Kitamura A and Yamada T (2021) Identification of *Faecalibacterium prausnitzii* strains for gut microbiome-based intervention in Alzheimer’s-type dementia. Cell Reports Medicine 2(9), 100398. 10.1016/j.xcrm.2021.100398 34622235 PMC8484692

[R186] van de Rest O, Berendsen AA, Haveman-Nies A and de Groot LC (2015) Dietary patterns, cognitive decline, and dementia: A systematic review. Advances in Nutrition 6(2), 154–168. 10.3945/an.114.007617 25770254 PMC4352174

[R187] van de Wouw M, Boehme M, Dinan TG and Cryan JF (2019) Monocyte mobilisation, microbiota & mental illness. Brain, Behavior, and Immunity 81, 74–91. 10.1016/j.bbi.2019.07.019 31330299

[R188] van der Lugt B, Rusli F, Lute C, Lamprakis A, Salazar E, Boekschoten MV, Hooiveld GJ, Muller M, Vervoort J, Kersten S, Belzer C, Kok DEG and Steegenga WT (2018) Integrative analysis of gut microbiota composition, host colonic gene expression and intraluminal metabolites in aging C57BL/6J mice. Aging (Albany NY) 10(5), 930–950. 10.18632/aging.101439 29769431 PMC5990381

[R189] Varma VR, Wang Y, An Y, Varma S, Bilgel M, Doshi J, Legido-Quigley C, Delgado JC, Oommen AM, Roberts JA, Wong DF, Davatzikos C, Resnick SM, Troncoso JC, Pletnikova O, O’Brien R, Hak E, Baak BN, Pfeiffer R, Baloni P, Mohmoudiandehkordi S, Nho K, Kaddurah-Daouk R, Bennett DA, Gadalla SM and Thambisetty M (2021) Bile acid synthesis, modulation, and dementia: A metabolomic, transcriptomic, and pharmacoepidemiologic study. PLoS Medicine 18(5), e1003615. 10.1371/journal.pmed.1003615 34043628 PMC8158920

[R190] Vogt NM, Kerby RL, Dill-McFarland KA, Harding SJ, Merluzzi AP, Johnson SC, Carlsson CM, Asthana S, Zetterberg H, Blennow K, Bendlin BB and Rey FE (2017) Gut microbiome alterations in Alzheimer’s disease. Scientific Reports 7(1), 13537. 10.1038/s41598-017-13601-y 29051531 PMC5648830

[R191] Wang J, Wei R, Xie G, Arnold M, Kueider-Paisley A, Louie G, Mahmoudian Dehkordi S, Blach C, Baillie R, Han X, De Jager PL, Bennett DA, Kaddurah-Daouk R and Jia W (2020) Peripheral serum metabolomic profiles inform central cognitive impairment. Scientific Reports 10(1), 14059. 10.1038/s41598-020-70703-w 32820198 PMC7441317

[R192] Wei ZY, Rao JH, Tang MT, Zhao GA, Li QC, Wu LM, Liu SQ, Li BH, Xiao BQ, Liu XY and Chen JH (2021) Characterization of changes and driver microbes in gut microbiota during healthy aging using a captive monkey model. Genomics Proteomics Bioinformatics 20, 350–365. 10.1016/j.gpb.2021.09.009 34974191 PMC9684162

[R193] Wieckowska-Gacek A, Mietelska-Porowska A, Wydrych M and Wojda U (2021) Western diet as a trigger of Alzheimer’s disease: From metabolic syndrome and systemic inflammation to neuroinflammation and neurodegeneration. Ageing Research Reviews 70, 101397. 10.1016/j.arr.2021.101397 34214643

[R194] Willmann C, Brockmann K, Wagner R, Kullmann S, Preissl H, Schnauder G, Maetzler W, Gasser T, Berg D, Eschweiler GW, Metzger F, Fallgatter AJ, Häring H-U, Fritsche A and Heni M (2020) Insulin sensitivity predicts cognitive decline in individuals with prediabetes. BMJ Open Diabetes Research & Care 8(2), e001741. 10.1136/bmjdrc-2020-001741 PMC7674089 33203727

[R195] Wilmanski T, Diener C, Rappaport N, Patwardhan S, Wiedrick J, Lapidus J, Earls JC, Zimmer A, Glusman G, Robinson M, Yurkovich JT, Kado DM, Cauley JA, Zmuda J, Lane NE, Magis AT, Lovejoy JC, Hood L, Gibbons SM, Orwoll ES and Price ND (2021) Gut microbiome pattern reflects healthy ageing and predicts survival in humans. Nature Metabolism 3(2), 274–286. 10.1038/s42255-021-00348-0 PMC8169080 33619379

[R196] Wilmanski T, Gibbons SM and Price ND (2022) Healthy aging and the human gut microbiome: Why we cannot just turn back the clock. Nature Aging 2(10), 869–871. 10.1038/s43587-022-00294-w 37118282 PMC10155257

[R197] Witte AV, Fobker M, Gellner R, Knecht S and Floel A (2009) Caloric restriction improves memory in elderly humans. Proceedings of the National Academy of Sciences of the United States of America 106(4), 1255–1260. 10.1073/pnas.0808587106 19171901 PMC2633586

[R198] Wu JW, Yaqub A, Ma Y, Koudstaal W, Hofman A, Ikram MA, Ghanbari M and Goudsmit J (2021a) Biological age in healthy elderly predicts aging-related diseases including dementia. Scientific Reports 11(1), 15929. 10.1038/s41598-021-95425-5 34354164 PMC8342513

[R199] Wu W-L, Adame MD, Liou C-W, Barlow JT, Lai T-T, Sharon G, Schretter CE, Needham BD, Wang MI, Tang W, Ousey J, Lin Y-Y, Yao T-H, Abdel-Haq R, Beadle K, Gradinaru V, Ismagilov RF and Mazmanian SK (2021b) Microbiota regulate social behaviour via stress response neurons in the brain. Nature 595(7867), 409–414. 10.1038/s41586-021-03669-y 34194038 PMC8346519

[R200] Wu L, Zeng T, Zinellu A, Rubino S, Kelvin DJ and Carru C (2019) A cross-sectional study of compositional and functional profiles of gut microbiota in sardinian centenarians. mSystems 4(4), e00325–19. 10.1128/mSystems.00325-19 31289141 PMC6616150

[R201] Xiang S, Ji JL, Li S, Cao XP, Xu W, Tan L and Tan CC (2022) Efficacy and safety of probiotics for the treatment of Alzheimer’s disease, mild cognitive impairment, and Parkinson’s disease: A systematic review and meta-analysis. Frontiers in Aging Neuroscience 14, 730036. 10.3389/fnagi.2022.730036 35185522 PMC8851038

[R202] Xiao J, Katsumata N, Bernier F, Ohno K, Yamauchi Y, Odamaki T, Yoshikawa K, Ito K and Kaneko T (2020) Probiotic *Bifidobacterium breve* in improving cognitive functions of older adults with suspected mild cognitive impairment: A randomized, double-blind, placebo-controlled trial. Journal of Alzheimers Disease 77(1), 139–147. 10.3233/JAD-200488 PMC7592675 32623402

[R203] Xue LJ, Yang XZ, Tong Q, Shen P, Ma SJ, Wu SN, Zheng JL and Wang HG (2020) Fecal microbiota transplantation therapy for Parkinson’s disease: A preliminary study. Medicine (Baltimore) 99(35), e22035. 10.1097/MD.0000000000022035 32871960 PMC7458210

[R204] Yang D, Zhao D, Ali Shah SZ, Wu W, Lai M, Zhang X, Li J, Guan Z, Zhao H, Li W, Gao H, Zhou X and Yang L (2019) The role of the gut microbiota in the pathogenesis of Parkinson’s disease. Frontiers in Neurology 10, 1155. 10.3389/fneur.2019.01155 31781020 PMC6851172

[R205] Zhao Y, Jaber V and Lukiw WJ (2017) Secretory products of the human GI tract microbiome and their potential impact on Alzheimer’s disease (AD): Detection of lipopolysaccharide (LPS) in AD hippocampus. Frontiers in Cellular and Infection Microbiology, 7, 318. 10.3389/fcimb.2017.00318 28744452 PMC5504724

